# Concentration–Dependent Effects of Exogenous α-Ketoglutarate on PSII Photochemical Performance and C–N Metabolism in *Carya cathayensis* under High Urea Application

**DOI:** 10.3390/plants15213286

**Published:** 2026-10-24

**Authors:** Jiaxin Hu, Yanyan Yin, Yutang Cheng, Bingsong Zheng, Daoliang Yan

**Affiliations:** State Key Laboratory for Development and Utilization of Forest Food Resources, Zhejiang A&F University, Hangzhou 311300, China

**Keywords:** α-ketoglutarate, *Carya cathayensis*, high urea application, carbon–nitrogen metabolism

## Abstract

Chinese hickory (*Carya cathayensis* Sarg.) is a significant woody oil tree species indigenous to China; nevertheless, excessive nitrogen (N) application has emerged as a primary limiting factor for sustainable production, as it induces leaf damage, metabolic imbalance, and premature senescence. α-Ketoglutarate (α-KG), an intermediate of the tricarboxylic acid (TCA) cycle, plays a crucial role in coordinating carbon (C) and N metabolism by providing carbon skeletons for N assimilation. In this study, one-year-old *C. cathayensis* seedlings were subjected to the following treatments: water control (CK), α-KG alone (40 mg/L, KG3), high urea (0.8%, U), and sequential applications of urea combined with 10, 20, or 40 mg/L α-KG (UKG1–UKG3). Photosynthetic characteristics, antioxidant responses, C–N metabolic parameters, and mineral element contents were evaluated at 0, 4, 8, and 12 weeks after treatment. High-concentration urea application led to visible leaf scorching and curling. At week 8, the urea-only treatment showed no significant difference from the water control in the six OJIP parameters; however, exogenous α-KG application significantly influenced the PSII fluorescence parameters of urea-treated seedlings. Compared with U, UKG3 markedly enhanced PSII performance, with the *PI_ABS_* value rising to 0.614 ± 0.093 (approximately 6.4 times that of U). Although urea treatment increased chlorophy ll content and net photosynthetic rate (*Pn*), these responses were not accompanied by a significant improvement in PSII photochemical efficiency relative to CK, indicating an imbalance between carbon assimilation and photosynthetic electron utilization under high urea supply. α-KG supplementation also improved nitrogen assimilation-related enzyme activities. GS activity under UKG treatments reached a level comparable to that of KG3. NR activity under UKG treatments decreased compared with both U and KG3 but remained higher than that of CK. α-KG alone enhanced photosynthetic performance, carbon accumulation, GS/Fd-GOGAT activities, and leaf Fe concentration without causing visible stress symptoms. Overall, exogenous α-KG supplementation partially alleviated high-urea-induced physiological disturbances in *C. cathayensis* seedlings, which was associated with improved PSII function and a more balanced regulation of C–N metabolism. These findings provide physiological evidence supporting the potential application of α-KG in optimizing nitrogen management strategies for *C. cathayensis* cultivation.

## 1. Introduction

Chinese hickory (*Carya cathayensis* Sarg.) is a woody oil and nut tree species endemic to China, with germplasm resources mainly distributed in the Tianmu Mountain region between Zhejiang and Anhui provinces [1,2]. In Lin’an District, Hangzhou City alone, its cultivation area has reached 570,000 mu (approximately 38,000 hectares), with an average annual nut output of about 17,000 tonnes and a total industrial chain output value of approximately 5 billion CNY [3,4]. The Chinese hickory industry has become a pillar of the regional agricultural economy and plays an important role in increasing the income of farmers in mountainous areas and promoting local ecological development.

Modern phytochemical studies have identified abundant bioactive compounds in the leaves of *C. cathayensis*, including cardamonin, pinostrobin chalcone, pinocembrin, chrysin, and wogonin. These flavonoids exhibit antioxidant and vascular-protective activities against H_2_O_2_-induced endothelial oxidative injury and cardiomyocyte hypoxia/reoxygenation damage [5,6]. In addition, characterization of the SCPL gene family in *C. cathayensis* revealed the role of SCPL4 in EGCG synthesis [7], further supporting the value of *C. cathayensis* leaves as a source of bioactive flavonoids and catechins.

In recent years, however, excessive nitrogen-based fertilizers have been applied indiscriminately to maximize short-term yield. This practice has led to continuous soil pH decline, shrinkage of the soil organic carbon pool, and severe imbalances in C and N stoichiometry [8,9,10]. Studies on Chinese agricultural soils have confirmed that long-term N addition significantly exacerbates soil acidification and affects soil organic carbon accumulation and turnover [11,12,13,14,15]. Consequently, long-term excessive N fertilization has become a primary factor responsible for yield reduction, premature tree senescence, and even mortality in *C. cathayensis* plantations, threatening the sustainable development of this industry [8].

Carbon serves as the core structural backbone of plants, with carbon (C) metabolism and nitrogen (N) metabolism representing two of the most fundamental and tightly coupled physiological processes [16,17,18]. Under high-N conditions, the amount of C fixed via photosynthesis often fails to meet the demand for carbon skeletons imposed by excessive N assimilation, making plants prone to carbon depletion or C–N imbalance [19].

α-Ketoglutarate (α-KG), a key intermediate of the tricarboxylic acid (TCA) cycle, lies at the intersection of C and N metabolism [20,21,22]. In the glutamine synthetase/glutamate synthase (GS/GOGAT) cycle, GS first catalyzes the adenosine triphosphate (ATP)-dependent condensation of glutamate with NH_4_^+^ to form glutamine, and ferredoxin-dependent glutamate synthase (Fd-GOGAT) subsequently transfers the amide group from glutamine to α-KG to yield two molecules of glutamate. Accordingly, α-KG functions as the carbon skeleton/acceptor in the Fd-GOGAT step, and each NH_4_^+^ assimilated through this cycle consumes one α-KG equivalent [23,24]. In addition, α-KG has been implicated in the regulation of nitrate assimilation, including the activity of nitrate reductase (NR) [23,24]. Therefore, the depletion of endogenous α-KG under high-N stress may act as a critical metabolic bottleneck that triggers and exacerbates carbon depletion.

Recent studies have demonstrated that exogenous α-KG can enhance antioxidant capacity and improve photosynthetic performance under saline–alkali, drought, and heavy metal stresses [25,26,27]. For instance, under nickel stress, exogenous α-KG alleviated photosynthetic damage and growth inhibition in *Peganum harmala* L. by strengthening antioxidant defense and promoting the synthesis of secondary metabolites [27]. Under heat stress, foliar application of α-KG increased chlorophyll content, photochemical efficiency, and membrane stability in perennial ryegrass (*Lolium perenne* L.) [28]. Under arsenic stress, α-KG improved seedling growth in eggplant (*Solanum melongena* L.) by enhancing the activities of NR, GS, and Fd-GOGAT and activating antioxidant defense [29]. In salt-stressed *Kosteletzkya virginica*, exogenous α-KG promoted C and N accumulation and optimized the C:N:P stoichiometric ratio [30]. Nevertheless, systematic research on whether and how exogenous α-KG alleviates high-N stress in *C. cathayensis* seedlings remains lacking.

In this study, one-year-old *C. cathayensis* seedlings were subjected to six treatments: a water control (CK), α-KG alone at 40 mg/L (KG3), high N (0.8% urea, U), and high N combined with α-KG at 10, 20, or 40 mg/L (UKG1, UKG2, UKG3). At 0, 4, 8, and 12 weeks after treatment, morphological traits, photosynthetic performance, antioxidant system parameters, key C/N metabolism enzyme activities, and microelement contents were measured. Using KG3 as a functional reference, this study aimed to clarify the synergistic alleviating effect of α-KG under high-N stress and provide a basis for scientific fertilization in *C. cathayensis* orchards.

## 2. Materials and Methods

### 2.1. Plant Material and Experimental Design

One-year-old healthy *C. cathayensis* Sarg. seedlings were grown in non-woven fabric containers (20 × 15 cm, diameter × height). The experiment was conducted in a plastic shade house with 50% shading and rain protection at Zhejiang A&F University, Hangzhou, China. The temperature and humidity were not controlled, and the photoperiod was natural daylight.

Six treatments were established: a water control (CK), 40 mg/L α-KG alone (KG3), high urea application (0.8%, U), and urea combined with three concentrations of α-KG (10, 20, and 40 mg/L; UKG1, UKG2, and UKG3, respectively). The treatment design is detailed in Table 1.

Each plant received 35 mL of the corresponding solution per application. All solutions were applied by foliar spraying until the leaf surfaces were uniformly wetted without visible runoff. Urea was applied on the first day of each treatment cycle, followed by α-KG application (or water application for treatments without α-KG) on the fourth day. A new treatment cycle was initiated after a 3-day interval. Treatments continued for 12 weeks.

At 4, 8, and 12 weeks after treatment initiation, the third and fourth compound leaves from the shoot apex were collected 4 days after α-KG application [31]. Samples were immediately frozen in liquid nitrogen and stored at −80 °C until physiological and biochemical analyses. A total of 90 plants were grown (6 treatments × 5 plants × 3 sampling times); at each sampling time, five plants per treatment were harvested destructively, and three were used as biological replicates. Week 0 samples were collected from the same batch of seedlings before treatment initiation.

### 2.2. Measurement Methods

All chemicals and reagents were of analytical grade and obtained from Sinopharm Chemical Reagent Co., Ltd. (Shanghai, China). Leaf area was measured using a YMJ-CHA3 intelligent leaf area meter (Zhejiang Top Cloud-Agri Technology Co., Ltd., Hangzhou, China). Leaves were oven-dried to constant weight for dry mass determination. Specific Leaf Area (SLA) was calculated as leaf area divided by dry mass, and Leaf Dry Matter Content (LDMC) was calculated as dry mass divided by fresh mass. Photosynthetic pigment contents were determined by extracting leaf discs in 95% ethanol for 48 h in the dark. The contents of chlorophyll a (Chl a), chlorophyll b (Chl b), and carotenoids were calculated using the modified Arnon equations of Lichtenthaler and Welburn [32].

Net photosynthetic rate (*Pn*) was measured using an LI-6800 portable photosynthesis system (LI-COR, Lincoln, NE, USA) under the following conditions: a CO_2_ concentration of 400 μmol/mol, a leaf temperature of 25 °C, a red/blue light ratio of 9:1, and a photosynthetically active radiation (PAR) of 1200 μmol m^−2^·s^−1^ [33].

Chlorophyll fluorescence induction kinetics (OJIP test) were measured at week 8 using a YZQ-500 modulated chlorophyll fluorometer (Yizongqi Technology Co., Ltd., Beijing, China). Leaves were dark-adapted for 30 min, then exposed to saturating blue actinic light at 3000 μmol photons m^−2^·s^−1^ for 5 s (saturation pulse, 800 ms). The OJIP parameters (*ABS/RC*, *φP*_0_, *Ψ*_0_, *φE*_0_, *φD*_0_, and *PI_ABS_*) were derived according to the JIP-test [34,35].

The activities of superoxide dismutase (SOD) and peroxidase (POD) were determined using the nitroblue tetrazolium (NBT) reduction method and the guaiacol oxidation method, respectively [36]. Fresh leaf samples (0.5 g) were homogenized in 50 mmol/L phosphate buffer (pH 7.8) and centrifuged at 6000 rpm for 30 min at 4 °C.

The soluble protein content was measured using the Coomassie Brilliant Blue G-250 method [37]. The contents of soluble sugar and starch were determined using the anthrone–sulfuric acid colorimetric method, with starch extracted using 52% perchloric acid at 25 °C for 30 min [38,39].

The activities of NR, GS, and Fd-GOGAT were determined using commercial assay kits (Suzhou Mengxi Biotechnology Co., Ltd., Suzhou, China; catalogue numbers M0203A, M0201A, and M0206A, respectively) following the manufacturer’s protocols.

The total nitrogen content was determined using the Kjeldahl method [40]. The concentrations of leaf Fe and Zn were determined using a Prodigy7 inductively coupled plasma optical emission spectrometer (Leeman, Hudson, NH, USA) following the multi-element analysis method [41]. Leaf samples were not washed before drying and digestion.

### 2.3. Statistical Analysis

Statistical analyses were performed using SPSS 26.0 (IBM Corp., Armonk, NY, USA). A two-way analysis of variance (ANOVA) was conducted to evaluate the effects of treatment, sampling time, and their interaction on measured parameters. When significant differences were detected, Tukey’s honestly significant difference (HSD) test was performed for multiple comparisons at *p* < 0.05. Different lowercase letters indicate significant differences among treatments at the same sampling time, whereas uppercase letters indicate significant differences among sampling times within the same treatment. For the urea + α-KG treatments, dose–response relationships were further evaluated using linear and quadratic regression analyses across α-KG concentrations of 10, 20, and 40 mg/L at each sampling time. The linear coefficient represents the change in response per 1 mg/L increase in α-KG concentration (Table S2). Significant linear and quadratic effects are indicated by asterisks where appropriate. Three biological replicates were used for each treatment, and all data are presented as mean ± standard deviation (SD). Detailed two-way ANOVA results are provided in Table S3.

## 3. Results

### 3.1. Effects of α-KG on Morphological Characteristics and Leaf Functional Traits Under High Urea Application

After 12 weeks of treatment, noticeable leaf scorching and curling symptoms were observed in seedlings treated with 0.8% urea (U). These symptoms were mitigated by sequential application of α-KG, particularly in the UKG2 and UKG3 treatments (Figure 1). Seedlings treated solely with α-KG (KG3) maintained normal leaf appearance throughout the experimental period.

High urea application (0.8% urea) slightly reduced the increase in plant height compared with CK (Figure 2), although the difference was not statistically significant. In contrast, α-KG supplementation partially restored height growth under high urea conditions. The increase in plant height exhibited a significant quadratic relationship with α-KG concentration, reaching a maximum under the UKG2 treatment (approximately 1.76-fold higher than that of U), followed by a slight decline under the UKG3 treatment. Additionally, the single application of KG3 resulted in a slightly greater height increment than CK, though the difference was relatively small.

During the treatment period, specific leaf area (SLA) and leaf dry matter content (LDMC) exhibited contrasting responses, as illustrated in Figure 3. SLA declined gradually over time across all treatments, though the magnitude of reduction varied among treatments. In KG3, SLA remained comparable to the control (CK) throughout the experiment. Under the U treatment, SLA declined significantly at 8 weeks, reaching 18.92 cm^2^/g at 12 weeks. The SLA values of UKG1, UKG2, and UKG3 were significantly lower than CK from week 8 onward and continued to decline during the treatment period.

In contrast, LDMC increased progressively with treatment duration. At weeks 0, 4, and 8, no significant differences in LDMC were observed between any treatment and CK. However, at week 12, the U treatment and all urea + α-KG treatments showed significantly higher LDMC than CK and KG3, with UKG3 exhibiting the highest LDMC value.

### 3.2. Photosynthetic Performance Under High Urea Application

#### 3.2.1. Photosynthetic Pigment Contents

The contents of chlorophyll *a*, chlorophyll *b*, and carotenoids exhibited distinct temporal patterns across treatments (Table 2).

Chlorophyll *a* content generally rose from week 0 to week 8 and remained stable at week 12. In the KG3 treatment, chlorophyll *a* content reached 2.06 ± 0.11 μg/mm^2^ at week 12, which was significantly higher than that in the CK treatment. Similar increasing trends were observed in the U and urea + α-KG treatments, where chlorophyll *a* contents were significantly higher than in the CK treatment at both weeks 8 and 12.

Chlorophyll *b* content showed relatively minor differences among treatments during the early growth stages. At week 12, the UKG3 treatment had the highest chlorophyll *b* content (1.06 ± 0.13 μg/mm^2^), which was significantly higher than that in the CK treatment, but did not differ significantly from the UKG1 and UKG2 treatments. The KG3 and U treatments also showed increased chlorophyll *b* contents compared with the CK treatment, although these differences were not statistically significant.

Carotenoid content increased in all treatments at week 4 and then declined subsequently. At week 8, the UKG1 treatment showed a significantly higher carotenoid content than the CK treatment, while no significant differences were detected among the other treatments. By week 12, carotenoid contents were comparable across all treatments.

#### 3.2.2. Chlorophyll Fluorescence Kinetic Parameters

Chlorophyll fluorescence parameters differed significantly among treatments at week 8 (Table 3). Relative to CK, KG3 showed lower *ABS/RC* and *φD*_0_ values and higher *φP*_0_, *Ψ*_0_, *φE*_0_, and *PI_ABS_* values.

Under 0.8% urea application (U), *PI_ABS_* showed the lowest numerical value among treatments (0.096 ± 0.015), although it was not significantly different from CK (0.137 ± 0.046). Similarly, no significant differences were detected between U and CK for *ABS/RC*, *φP*_0_, *Ψ*_0_, *φE*_0_, and *φD*_0_. However, compared with KG3 and the urea + α-KG treatments, U exhibited significantly lower *φP*_0_, *Ψ*_0_, *φE*_0_, and *PI_ABS_* values, accompanied by higher *ABS/RC* and *φD*_0_ values.

PSII fluorescence parameters showed concentration-dependent responses to α-KG addition under high urea conditions. Regression analysis showed significant linear and quadratic responses of the evaluated parameters to α-KG concentration (*p* < 0.05 or *p* < 0.001). *ABS/RC* and *φD*_0_ showed significant decreasing trends with increasing α-KG concentration, whereas *φP*_0_, *Ψ*_0_, *φE*_0_, and *PI_ABS_* showed significant increasing trends. Among the urea + α-KG treatments, *PI_ABS_* increased progressively with α-KG concentration, reaching 0.371 ± 0.065, 0.490 ± 0.049, and 0.614 ± 0.093 in UKG1, UKG2, and UKG3, respectively, representing approximately 3.9-, 5.1-, and 6.4-fold increases relative to U. Under UKG3 treatment, *ABS/RC* and *φD*_0_ decreased to 3.595 ± 0.201 and 0.310 ± 0.003, respectively, whereas *φP*_0_, *Ψ*_0_, and *φE*_0_ increased to 0.690 ± 0.003, 0.496 ± 0.023, and 0.342 ± 0.017, respectively.

#### 3.2.3. Net Photosynthetic Rate

As shown in Figure 4, the net photosynthetic rate (*Pn)* dynamics varied significantly among the different treatments. At week 0, no significant differences in *Pn* were observed across treatments. Under the CK (control) treatment, *Pn* remained relatively stable with minor fluctuations throughout the experimental period. Under the KG3 treatment, *Pn* was significantly higher than that of CK at week 4 (4.05 ± 0.35 vs. 3.21 ± 0.13 μmol CO_2_·m^−2^·s^−1^) and peaked at 5.63 ± 0.45 μmol CO_2_·m^−2^·s^−1^ by week 8, a value slightly higher than that of the U treatment ((5.46 ± 0.18 μmol CO_2_·m^−2^·s^−1^). Although *Pn* in the KG3 treatment decreased from week 8 to week 12, it remained significantly higher than that of CK and comparable to U. These results indicate that α-ketoglutarate (α-KG) alone can function as a photosynthetic promoter under normal cultivation conditions, with the most pronounced promotional effect observed at week 8.

Under urea alone, *Pn* was significantly elevated at weeks 4, 8, and 12 relative to CK, indicating that high-concentration urea treatment alone stimulated photosynthesis. However, this was accompanied by leaf scorch, and *Pn* declined at week 12. When α-KG was applied in combination, *Pn* was further enhanced at weeks 4 and 8 in a dose-dependent manner. Regression analysis revealed a significant linear dose effect at week 4 (*p* < 0.001, linear ↑), and significant linear and quadratic dose effects at week 8 (linear *p* = 0.025; quadratic *p* = 0.006), whereas no significant dose effect was observed at week 0 or week 12. At the same time points, the sequential application groups generally showed numerically higher *Pn* than U alone and mostly higher *Pn* than KG3 alone. For UKG3, *Pn* reached 6.07 ± 0.06 μmol CO_2_·m^−2^·s^−1^ at week 4, representing increases of 20.4% over U and 49.9% over KG3 (4.05 ± 0.35 μmol CO_2_·m^−2^·s^−1^). At week 8, *Pn* under UKG3 remained high at 6.17 ± 0.06 μmol CO_2_·m^−2^·s^−1^; at week 12, although it decreased to 5.26 ± 0.35 μmol CO_2_·m^−2^·s^−1^, it remained significantly higher than CK, while being comparable to KG3 and U.

### 3.3. Antioxidant Responses Under High Urea Application

#### 3.3.1. SOD and POD Activities

After 12 weeks of treatment, antioxidant enzyme activities exhibited distinct responses across the different treatments (Figure 5). SOD activity in KG3 was significantly higher than that in CK, whereas POD activity was significantly lower than in CK. Under the U treatment, both SOD and POD activities were significantly increased compared with CK. The sequential application of α-KG in combination with urea altered antioxidant enzyme activities relative to U alone. SOD activity in all UKG treatments was lower than in U, and exhibited a decreasing trend with increasing α-KG concentration. However, SOD activities in UKG treatments remained significantly higher than in CK. POD activity showed a similar response pattern. All UKG treatments exhibited significantly lower POD activity than U. With increasing α-KG concentration, POD activity gradually declined and approached the level observed in CK. No significant difference was observed between UKG2 and UKG3.

#### 3.3.2. Soluble Protein Content

The soluble protein content changed dynamically throughout the treatment period (Figure 6). At week 4, all treatments exhibited significantly higher soluble protein contents than the control (CK), with no significant differences detected among the treatments themselves. At week 8, the soluble protein content in KG3 rose to 16.60 ± 2.43 mg/g. This value was not significantly different from that of the U treatment (16.03 ± 4.59 mg/g), but both were significantly higher than CK. The UKG treatments demonstrated similar soluble protein levels, showing no significant differences among them. At week 12, the soluble protein content in U remained significantly higher than both CK and KG3 (13.39 ± 1.46 vs. 8.19 ± 1.43 mg/g). Among the urea + α-KG treatments, soluble protein content increased with rising α-KG concentrations, although the differences among the UKG treatments were relatively minor.

### 3.4. Carbon and Nitrogen Metabolism Under High Urea Application

#### 3.4.1. Soluble Sugar Content

Soluble sugar content serves as an indicator of plant carbon metabolic status (Figure 7). At week 0, no significant differences were detected among treatments. At week 4, CK exhibited a slight decline compared with week 0 (75.68 mg/g), whereas all other groups increased. UKG3 recorded the highest value, reaching 121.19 mg/g, which was significantly higher than CK, KG3, and U, but not significantly different from UKG1 or UKG2. At week 8, U, UKG2, and UKG3 maintained relatively high soluble sugar levels and were significantly higher than CK, whereas KG3 and UKG1 were not significantly different from CK. This suggests that the sequential application of urea and α-KG promoted soluble sugar accumulation in the early phase. At week 12, KG3 still maintained a high level of 94.78 mg/g. In contrast, all urea-containing treatments (U, UKG1, UKG2, and UKG3) decreased sharply to 51.84, 56.97, 62.70, and 53.69 mg/g, respectively. These values were significantly lower than CK at the same time point and the initial level at week 0. This pattern of initial accumulation followed by a sharp decline may be attributed to the massive consumption of carbon skeletons during sustained urea assimilation.

#### 3.4.2. Starch Content

Leaf starch content exhibited distinct dynamics with increasing treatment duration (Figure 8). At week 0, starch content did not differ significantly among treatments. At week 4, starch in CK increased slightly, whereas it decreased numerically in KG3, UKG1, and UKG2 and remained relatively stable in U and UKG3; however, no significant differences were detected among treatments. At week 8, starch in CK and KG3 increased sharply to peak values of 9.87 and 9.18 mg/g, respectively, representing increases of approximately 160% and 135% compared with week 0. At this time point, starch in U and its combination treatments (UKG1, UKG2, and UKG3) was significantly lower than in CK and KG3. By week 12, CK and KG3 decreased significantly from their week 8 peaks, whereas U did not change significantly; UKG2 and UKG3 steadily accumulated starch to their highest values during the experiment. Thus, CK and KG3 displayed an initial increase followed by a decline, while sequential application of U with high concentrations of α-KG (UKG2 and UKG3) was characterized by sustained starch accumulation.

#### 3.4.3. Activities of Key Nitrogen Metabolism Enzymes

At week 8, GS, Fd-GOGAT, and NR activities differed among treatments (Figure 9). GS activity in CK was 8.84 ± 0.15 μmol/g/h. Compared with CK, KG3 showed significantly higher GS activity (13.65 ± 0.12 μmol/g/h), whereas U showed an intermediate value (11.31 ± 0.20 μmol/g/h), which was significantly higher than CK but lower than KG3. Under urea + α-KG treatments, GS activities in UKG1, UKG2, and UKG3 were 13.31 ± 0.06, 13.40 ± 0.06, and 13.59 ± 0.29 μmol/g/h, respectively. All UKG treatments showed significantly higher GS activity than U and no significant differences from KG3.

Fd-GOGAT activity under KG3 was significantly higher than CK (174.90 ± 3.47 vs. 134.96 ± 4.16 nmol/min/g), whereas no significant difference was detected between KG3 and U (155.45 ± 17.09 nmol/min/g). Under urea + α-KG treatments, Fd-GOGAT activity was significantly higher than CK, but no significant differences were observed among UKG1 (160.64 ± 5.53), UKG2 (163.23 ± 5.14), and UKG3 (166.15 ± 1.68 nmol/min/g). These values were statistically similar to those observed in U and KG3.

NR activity showed a different response pattern from GS and Fd-GOGAT. NR activity in KG3 (207.78 ± 9.74 nmol/min/g) and U (218.62 ± 8.90 nmol/min/g) was significantly higher than CK (115.74 ± 12.86 nmol/min/g), with U showing the highest value among treatments. No significant difference was detected between U and KG3. Under urea + α-KG treatments, NR activities in UKG1, UKG2, and UKG3 were 144.12 ± 2.30, 156.05 ± 10.37, and 149.75 ± 7.19 nmol/min/g, respectively. All UKG treatments showed significantly lower NR activity than U and KG3, while remaining higher than CK.

#### 3.4.4. Dynamic Changes in Total Nitrogen Content

Total nitrogen content (Figure 10) showed an overall increasing trend over time in all treatments. At week 0, total N did not vary significantly among treatments. In CK, total N rose steadily from 11.45 ± 0.57 mg/g at week 0 to 15.41 ± 0.29 mg/g at week 12, an increase of approximately 34.5%. At week 4, total N in all treatment groups was significantly higher than CK, with the largest increase in UKG2. By week 8, total N continued to increase substantially in all treatments, while CK remained significantly the lowest. At week 12, total N reached peak values. U (30.35 ± 7.00 mg/g) and UKG2 (30.31 ± 0.03 mg/g) exhibited the most pronounced accumulation, with increases of 159.6% and 171.1%, respectively, relative to week 0. Both were significantly higher than UKG1, while U and UKG2 did not differ significantly from UKG3.

Regression analysis further revealed the dose-dependent pattern of total N in response to α-KG under urea. At week 0, neither the linear nor the quadratic term was significant (*p* > 0.05). At weeks 4, 8, and 12, the linear terms were not significant (*p* = 0.237, 0.231, and 0.931, respectively), whereas the quadratic terms were significant (*p* = 0.004, 0.002, and <0.001, respectively), indicating a significant quadratic dose response at these time points. These results suggest that urea alone or urea combined with a moderate concentration of α-KG (UKG2) was associated with late-stage N accumulation in leaves, whereas high-concentration α-KG (UKG3) showed numerically lower total N than U and UKG2, although the difference was not statistically significant.

### 3.5. Leaf Fe and Zn Concentrations Under High Urea Application

Leaf Fe and Zn concentrations were measured after 12 weeks of treatment to evaluate the effects of urea and α-KG application on mineral element accumulation. Significant differences in Fe concentration were observed among treatments (Figure 11). Compared with CK (214.05 ± 12.60 mg/kg), KG3 significantly increased leaf Fe concentration to 298.59 ± 15.96 mg/kg. Urea application alone also enhanced Fe concentration (267.11 ± 21.84 mg/kg), which was significantly higher than that of CK. Under the combined urea and α-KG treatments, Fe concentrations in UKG1, UKG2, and UKG3 were all significantly higher than that of CK, but no significant differences were observed among the three α-KG application levels.

Zn concentration showed a distinct response pattern compared with Fe. Under CK conditions, leaf Zn concentration was 197.27 ± 8.67 mg/kg, and KG3 treatment had no significant effect on Zn accumulation (219.70 ± 16.99 mg/kg). Urea treatment significantly increased leaf Zn concentration to 273.91 ± 22.75 mg/kg compared with CK. Under high-N conditions, the addition of α-KG significantly reduced leaf Zn concentrations in UKG1 and UKG2 compared with U, with UKG1 decreasing to 205.27 ± 17.86 mg/kg. However, Zn concentration increased gradually with increasing α-KG concentration, reaching 281.86 ± 13.73 mg/kg in UKG3, which was not significantly different from that under U.

## 4. Discussion

### 4.1. Effects of α-KG Addition on PSII Photochemical Performance Under Urea Treatment

Excessive urea application increased chlorophyll *a* content and net photosynthetic rate (*Pn*) in *C. cathayensis* leaves, consistent with an early N-stimulation phase in which enhanced N availability promotes chloroplast development and photosynthetic protein synthesis [42,43]. Nevertheless, visible leaf scorching and curling were observed, consistent with known foliar urea phytotoxicity [44,45,46].

At week 8, no significant differences were observed between U and CK in the six chlorophyll fluorescence parameters, including *ABS/RC*, *φP*_0_, *Ψ*_0_, *φE*_0_, and *φD*_0_ (Table 3). However, *PI_ABS_* under U was the lowest among treatments (0.096 ± 0.015), whereas that under UKG3 was approximately 6.4-fold higher, indicating a substantial decline in overall PSII performance. Since *φP*_0_ mainly reflects the maximum photochemical efficiency of active reaction centres and is relatively insensitive to changes in electron transport capacity [47], the unchanged *φP*_0_ under U does not necessarily indicate unaffected PSII function. Instead, the reduction in *PI_ABS_* suggests that excessive urea may primarily impair downstream electron transport processes, particularly the acceptor-side reactions beyond QA^−^, rather than causing complete photoinactivation of PSII reaction centres [48].

α-KG supplementation improved PSII performance in a concentration-dependent manner without significantly altering total chlorophyll content. Under UKG3, *PI_ABS_* increased approximately 6.4-fold compared with U (0.614 ± 0.093 vs. 0.096 ± 0.015), accompanied by an increase in *φE*_0_. Similar protective effects of α-KG have been reported under other abiotic stresses. In arsenate-stressed eggplant, α-KG alleviated reductions in primary photochemistry, electron transport efficiency, and *PI_ABS_* while enhancing N-assimilation-related enzymes, including NR, nitrite reductase (NiR), GS, and GOGAT [29]. In cold-stressed soybean, exogenous α-oxoglutarate improved photosynthetic performance, increased GS activity, and reduced ammonium accumulation [49]. Consistent with the present findings, previous studies have also reported a stronger response under higher α-KG concentrations, further supporting a concentration-dependent effect of α-KG on PSII regulation.

Interestingly, KG3 alone maintained relatively high *PI_ABS_* and *φE*_0_, and no significant difference was detected between KG3 and UKG3. This indicates that the beneficial effects of α-KG on PSII were not solely attributable to the mitigation of urea toxicity but may also involve regulation of chloroplast carbon–nitrogen metabolism. In illuminated chloroplasts, the GS/GOGAT cycle consumes reducing equivalents generated by photosynthetic electron transport and uses α-KG as the carbon skeleton for ammonium assimilation [50]. Thus, sufficient α-KG availability may facilitate electron utilisation by enhancing ammonium assimilation, thereby maintaining QA oxidation and reducing excessive excitation pressure on PSII. As a central intermediate of the TCA cycle, α-KG links carbon availability with nitrogen metabolism and may contribute to maintaining PSII acceptor-side electron flow under excessive N supply [24,51,52]. Nevertheless, direct measurements of endogenous α-KG pools and electron transport components are required to further validate this mechanism.

### 4.2. Effects of α-KG Addition on Carbon Metabolism Under Urea Treatment

Urea application induced a dynamic shift in carbon metabolism in *C. cathayensis* seedlings. During the early stage (4 weeks), U-treated plants maintained relatively high soluble sugar and starch contents, suggesting that enhanced nitrogen (N) availability initially promoted carbon assimilation and allocation. This response is consistent with the early anabolic phase of N stimulation, during which increased N supply boosts photosynthetic capacity and supports the synthesis of N-containing compounds [53]. However, by week 12, soluble sugar and starch contents declined markedly under the U treatment, with soluble sugar levels significantly lower than those in the control (CK). This temporal shift indicates that prolonged excessive urea supply altered carbon allocation patterns rather than simply inhibiting photosynthetic carbon fixation.

The reduction in carbohydrate reserves under the U treatment was likely linked to increased carbon demand for N assimilation. Excessive N availability stimulates ammonium incorporation and raises the consumption of carbon skeletons for amino acid biosynthesis, thereby reducing carbon storage compounds [54,55]. Similar reductions in non-structural carbohydrates and starch have been documented in apple trees and Norway spruce under excessive N supply, where carbon was preferentially allocated to N assimilation rather than storage [56,57]. A meta-analysis further confirmed that N addition generally lowers foliar non-structural carbohydrate concentrations, supporting the view that excessive N can disrupt carbon balance by increasing metabolic demand [53]. Thus, the late-stage decline in soluble sugar and starch under the U treatment likely reflects enhanced carbon utilization for N assimilation rather than a direct limitation of photosynthetic carbon acquisition. Nevertheless, changes in biomass accumulation and whole-plant carbon flux remain to be investigated.

α-Ketoglutarate (α-KG) supplementation modified this carbon allocation pattern under excessive urea conditions. Between weeks 4 and 8, the UKG3 treatment had the highest soluble sugar content among all groups, indicating improved carbon retention during the period of active N assimilation. As a key carbon skeleton for glutamine synthetase/glutamate synthase (GS/GOGAT)-mediated ammonium assimilation, α-KG may alleviate carbon skeleton limitation and reduce the diversion of newly fixed carbon into N assimilation pathways [24,58]. At week 12, starch content under the U treatment was significantly lower than under the KG3 and UKG3 treatments, while UKG3 maintained starch levels comparable to those of the CK and KG3 groups. This recovery suggests that starch depletion under the U treatment was associated with increased carbon extraction for N assimilation, and that α-KG supplementation mitigated this demand by supplying additional carbon skeletons [54,55].

The divergent responses between the KG3 and UKG treatments further demonstrate that α-KG regulates carbon metabolism in a concentration-dependent manner. The KG3 treatment consistently maintained relatively high soluble sugar and starch contents. The UKG2 and UKG3 treatments showed comparable abilities to preserve soluble sugar and starch reserves at weeks 4 and 8. By week 12, UKG2 exhibited a slight numerical advantage in soluble sugar retention, whereas UKG3 maintained a comparable starch level. These minor differences suggest that both 20 mg/L and 40 mg/L α-KG provide sufficient carbon skeletons to buffer excessive N assimilation, with the slight variations reflecting temporal dynamics rather than distinct functional roles.

### 4.3. Effects of α-KG Addition on Nitrogen Assimilation Under Urea Treatment

Nitrogen assimilation is closely coupled with carbon metabolism through the GS/GOGAT cycle, in which α-KG provides the carbon skeleton required for ammonium incorporation into amino acids [59,60]. Under excessive urea supply, increased N availability enhances the demand for carbon skeletons, and insufficient α-KG availability may restrict efficient ammonium assimilation. This balance is particularly important in woody plants, where GS-mediated ammonium reassimilation represents a major pathway for maintaining nitrogen homeostasis [59].

In the present study, KG3 significantly increased GS and Fd-GOGAT activities compared with CK, indicating that exogenous α-KG promoted the enzymatic capacity associated with N assimilation rather than acting solely as a metabolic carbon source. This finding is consistent with previous studies showing that 2-oxoglutarate supplementation enhances GS, NADH-dependent glutamate synthase (NADH-GOGAT), phosphoenolpyruvate carboxylase (PEPC) and NADP-dependent isocitrate dehydrogenase (NADP-ICDH) activities, promotes ammonium assimilation and increases glutamine/glutamate accumulation in rice [58]. Under U treatment, GS activity increased compared with CK, yet it remained significantly lower than that under KG3 and all UKG treatments. This suggests that although excessive urea provided abundant N, GS activity may have been constrained by limited carbon skeleton availability. Exogenous α-KG could therefore alleviate this metabolic limitation by increasing substrate availability for ammonium assimilation. Similar substrate-dependent regulation has been observed in wheat cells, where ammonium excess reduced α-KG pools and altered GS activity through changes in the balance among ammonium, glutamine and α-KG [60].

The response of Fd-GOGAT differed from that of GS. Although Fd-GOGAT activity increased under U compared with CK, α-KG supplementation did not further enhance Fd-GOGAT activity relative to U, while all UKG treatments remained higher than CK. This difference may reflect the distinct regulatory characteristics of these two enzymes. In *Arabidopsis*, leaf Fd-GOGAT is strongly associated with light-dependent photorespiratory ammonium reassimilation, whereas GS represents the primary entry point for ammonium incorporation into organic nitrogen compounds [61]. Because Fd-GOGAT activity depends largely on ferredoxin availability and photosynthetic electron supply, its response may be less directly regulated by α-KG availability than GS under the present conditions. Thus, GS appears to represent the more responsive step linking α-KG supply with enhanced nitrogen assimilation.

Total leaf N increased in all treatments during the 12-week experiment, with urea-treated plants showing greater N accumulation than those in the CK treatment. However, the increased N content under U was accompanied by reduced soluble sugar and starch levels, indicating that N acquisition exceeded the capacity for carbon-supported assimilation. In contrast, α-KG supplementation improved the coordination between N accumulation and carbon metabolism. For example, UKG2 maintained higher soluble sugar and protein contents despite having a total N level comparable with U, suggesting that α-KG promoted the incorporation of absorbed N into organic compounds rather than simply increasing N accumulation [49,58]. This interpretation is consistent with the central role of α-KG as a metabolic signal connecting carbon availability with nitrogen assimilation [24].

The soluble protein content in the KG3 treatment peaked at week 8 and then declined sharply by approximately 51% by week 12, which may be related to the absence of a continuous exogenous N supply. By providing carbon skeletons, α-KG accelerated the conversion of leaf-assimilable N into soluble protein during the early treatment phase; as this finite endogenous N pool was rapidly depleted, carbon skeletons remained abundant in the later stage, whereas de novo protein synthesis became limited by the shortage of N substrates. This contrasts with UKG2, which maintained higher soluble protein levels under continuous N supply, further indicating that α-KG functions to promote the incorporation of absorbed N into organic compounds rather than to increase total N accumulation.

NR activity showed a different response pattern. Compared with U and KG3, NR activity was reduced under urea + α-KG treatments but remained higher than that of CK. This decrease may reflect feedback regulation of nitrate reduction after enhanced GS/GOGAT activity and increased organic N synthesis reduced the demand for additional N assimilation flux [60]. However, because inorganic N forms and amino acid pools were not measured, the precise regulatory mechanism remains to be confirmed. Overall, these results indicate that α-KG improves nitrogen assimilation efficiency under excessive urea conditions primarily by enhancing carbon-supported ammonium incorporation rather than by simply increasing total N accumulation.

### 4.4. Effects of α-KG Addition on Antioxidant Defense and Leaf Fe/Zn Concentrations Under Urea Treatment

Excessive urea application disturbed oxidative homeostasis in *C. cathayensis*, as indicated by increased SOD and POD activities under U treatment. Enhanced antioxidant enzyme activity generally represents an induced defence response to elevated reactive oxygen species (ROS) production rather than an indicator of improved physiological status. Similar responses have been reported under urea-induced stress, where increased ROS accumulation and lipid peroxidation were accompanied by activation of antioxidant enzymes [62]. In the present study, the occurrence of leaf scorching and curling despite elevated SOD/POD activities suggests that antioxidant activation was insufficient to fully prevent cellular damage. Such injury may have resulted from multiple effects of excessive foliar urea, including osmotic stress, direct tissue damage and potential ammonium/ammonia toxicity following urea hydrolysis [44,45,46].

α-KG supplementation, particularly UKG3, alleviated visible leaf injury and reduced SOD/POD activities compared with U. The lower antioxidant enzyme activities under UKG3 likely reflect reduced oxidative pressure rather than weakened defence capacity. Improved PSII electron transport, as indicated by increased *PI_ABS_* and *φE*_0_, may have reduced excessive excitation energy and electron leakage to oxygen, thereby limiting ROS generation at the photosynthetic level [29]. In addition, α-KG itself may contribute to oxidative protection through direct non-enzymatic scavenging of H_2_O_2_, producing succinate, CO_2_ and water [63]. Thus, α-KG-mediated protection may involve both improved regulation of photosynthetic electron flow and direct chemical ROS detoxification.

Changes in leaf Fe concentration suggest a possible association between α-KG-mediated metabolic regulation and Fe-related physiological processes. KG3 increased leaf Fe concentration and Fd-GOGAT activity, whereas UKG3 maintained Fe levels significantly higher than CK and comparable with U. Iron is essential for photosynthetic electron transport, ferredoxin-dependent Fd-GOGAT activity and several Fe-containing antioxidant systems [61,64]. Therefore, the concurrent improvement of Fe status, Fd-GOGAT activity and PSII performance under α-KG treatment is consistent with enhanced Fe-dependent metabolic function. However, total leaf Fe concentration alone cannot distinguish changes in Fe uptake, translocation, storage or incorporation into functional Fe proteins. Further analysis of ferredoxin abundance, Fe-containing proteins and Fe subcellular distribution will be required to determine whether Fe-related processes contribute directly to α-KG-mediated stress mitigation. Furthermore, because the leaves were repeatedly foliar-sprayed and not washed before analysis, the potential contribution of surface contamination to the measured leaf Fe and Zn concentrations cannot be excluded.

Overall, α-KG supplementation reduced oxidative stress under excessive urea mainly by maintaining PSII electron transport and improving C–N metabolic coordination, rather than by enhancing antioxidant enzyme activation alone.

## 5. Conclusions

Exogenous α-KG supplementation partially mitigated the physiological disturbances induced by excessive urea application in *C. cathayensis* seedlings, as evidenced by reduced leaf injury symptoms, improved PSII photochemical performance, and enhanced coordination between C and N metabolism. The responses to α-KG were concentration-dependent and varied among physiological processes.

The high-concentration α-KG treatments (UKG2 and UKG3) exhibited broadly similar dynamic patterns in carbohydrate accumulation, suggesting that both 20 and 40 mg/L α-KG supply adequate carbon skeletons to buffer excessive N assimilation. α-KG alone also promoted photosynthetic performance and GS/GOGAT-related N assimilation without causing visible adverse effects, suggesting that α-KG may function as a metabolic regulator in *C. cathayensis* seedlings.

Although these results demonstrate the beneficial physiological effects of α-KG supplementation, the specific roles of ammonium metabolism, endogenous α-KG availability, and Fe homeostasis remain to be clarified, and unwashed leaf surfaces may have contributed to the measured Fe and Zn concentrations. Moreover, repeated foliar urea application may have contributed to leaf injury independently of systemic N responses. Future studies integrating inorganic N profiling, metabolomic analysis, ROS characterization, and Fe-related regulatory pathways will help elucidate the mechanisms underlying α-KG-mediated adaptation to excessive urea application.

## Figures and Tables

**Figure 1:**
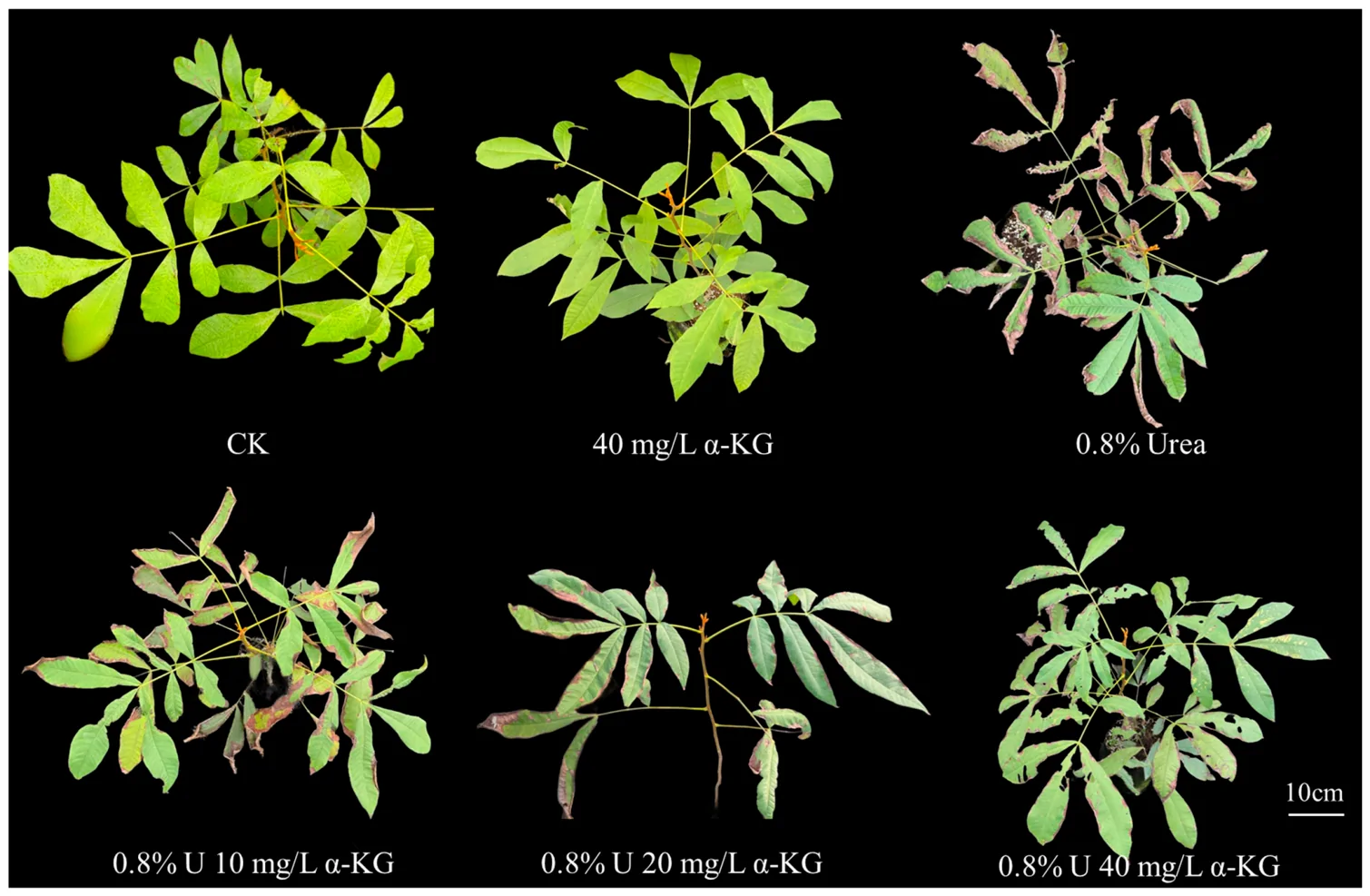
Phenotypes of Chinese hickory (*C. cathayensis* Sarg.) seedlings after 12 weeks of treatment.

**Figure 2:**
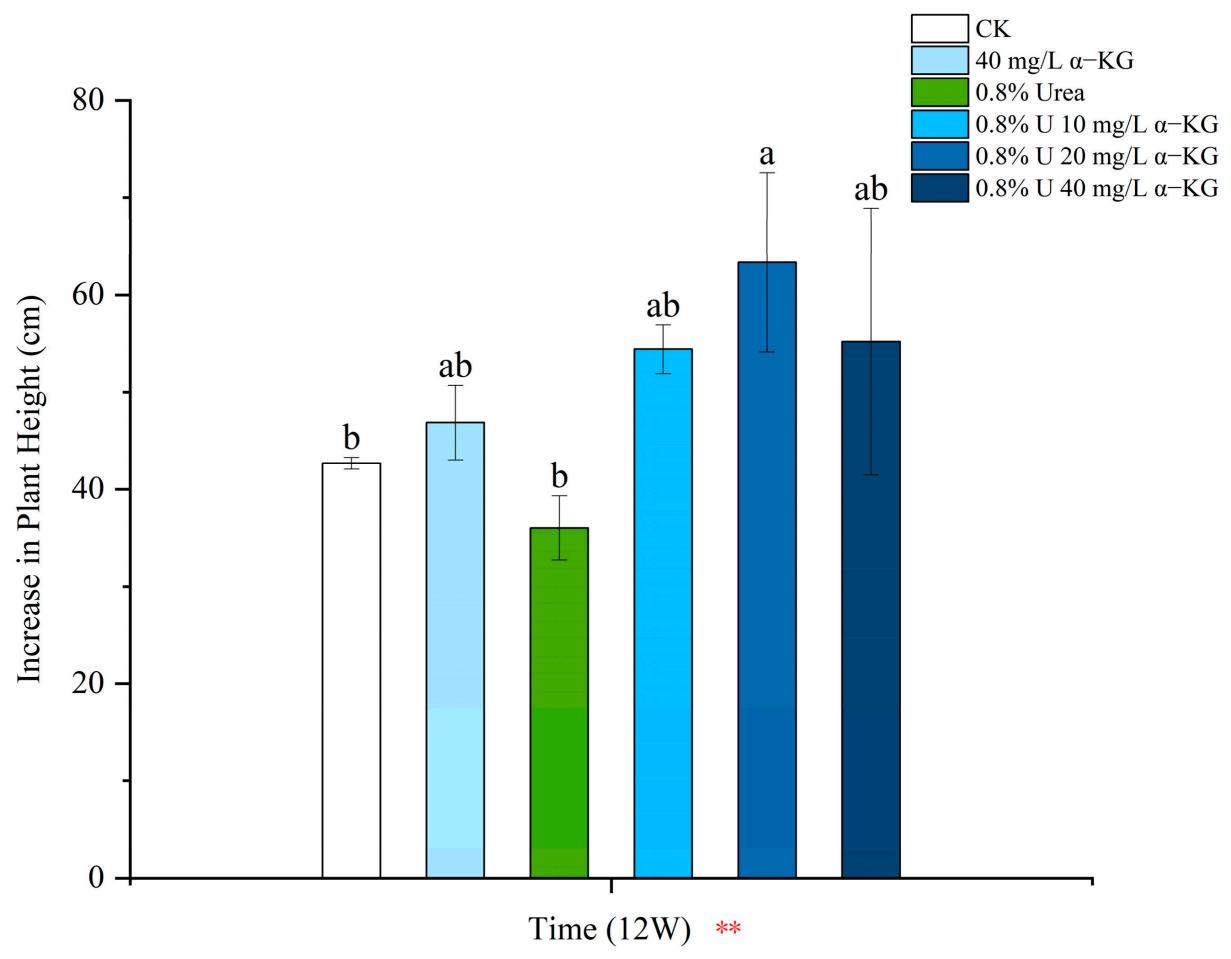
Plant height increment of Chinese hickory (*C. cathayensis* Sarg.) seedlings after 12 weeks of treatment. Note: Different letters indicate significant differences among treatments (one-way ANOVA followed by Tukey’s HSD test, *p* < 0.05). Values are means ± SD. Red asterisks indicate significant quadratic trends across U + α-KG doses (10, 20, 40 mg/L) at each sampling time. ** *p* < 0.01.

**Figure 3:**
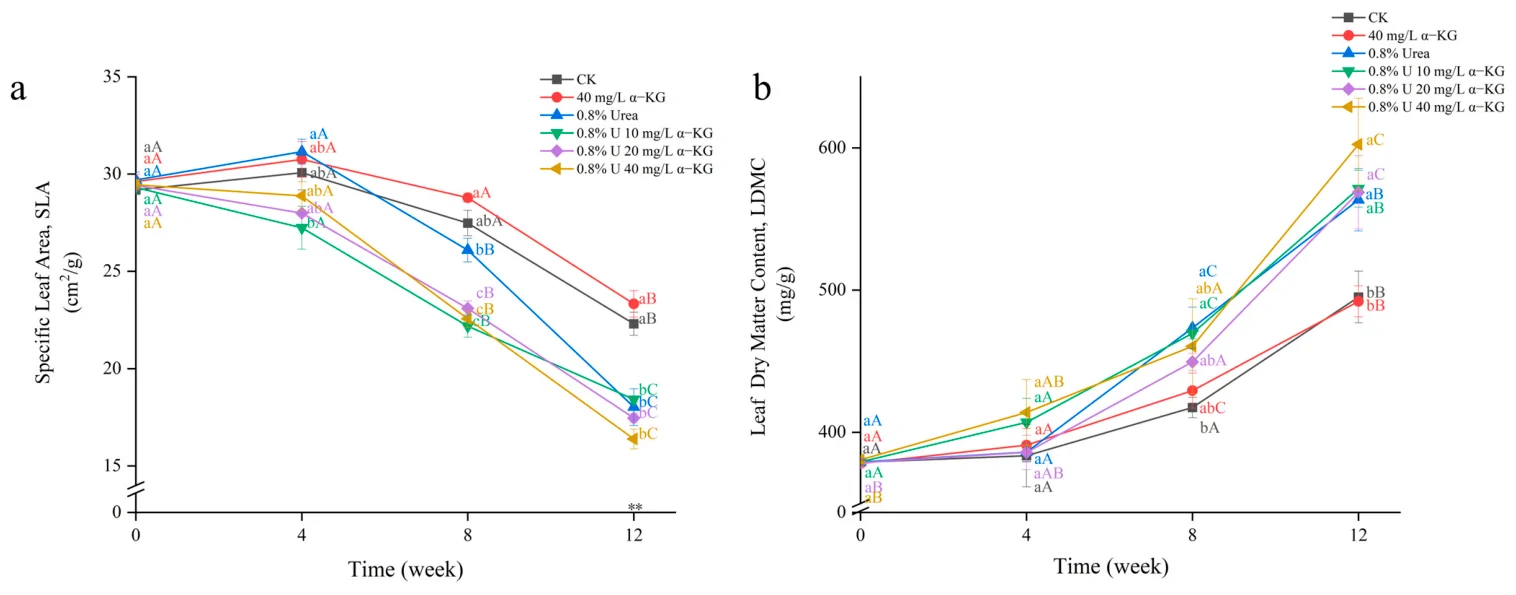
Specific leaf area (SLA, cm^2^/g; (**a**) and leaf dry matter content (LDMC, mg/g; (**b**) of Chinese hickory (*C. cathayensis* Sarg.) leaves under different treatments at different sampling times. Note: Different lowercase letters indicate significant differences among treatments within the same time point, and different uppercase letters indicate significant differences among time points within the same treatment (two-way ANOVA followed by Tukey’s HSD test, *p* < 0.05). Black asterisks indicate significant linear trends. ** *p* < 0.01. Error bars represent mean ± SD (*n* = 3).

**Figure 4:**
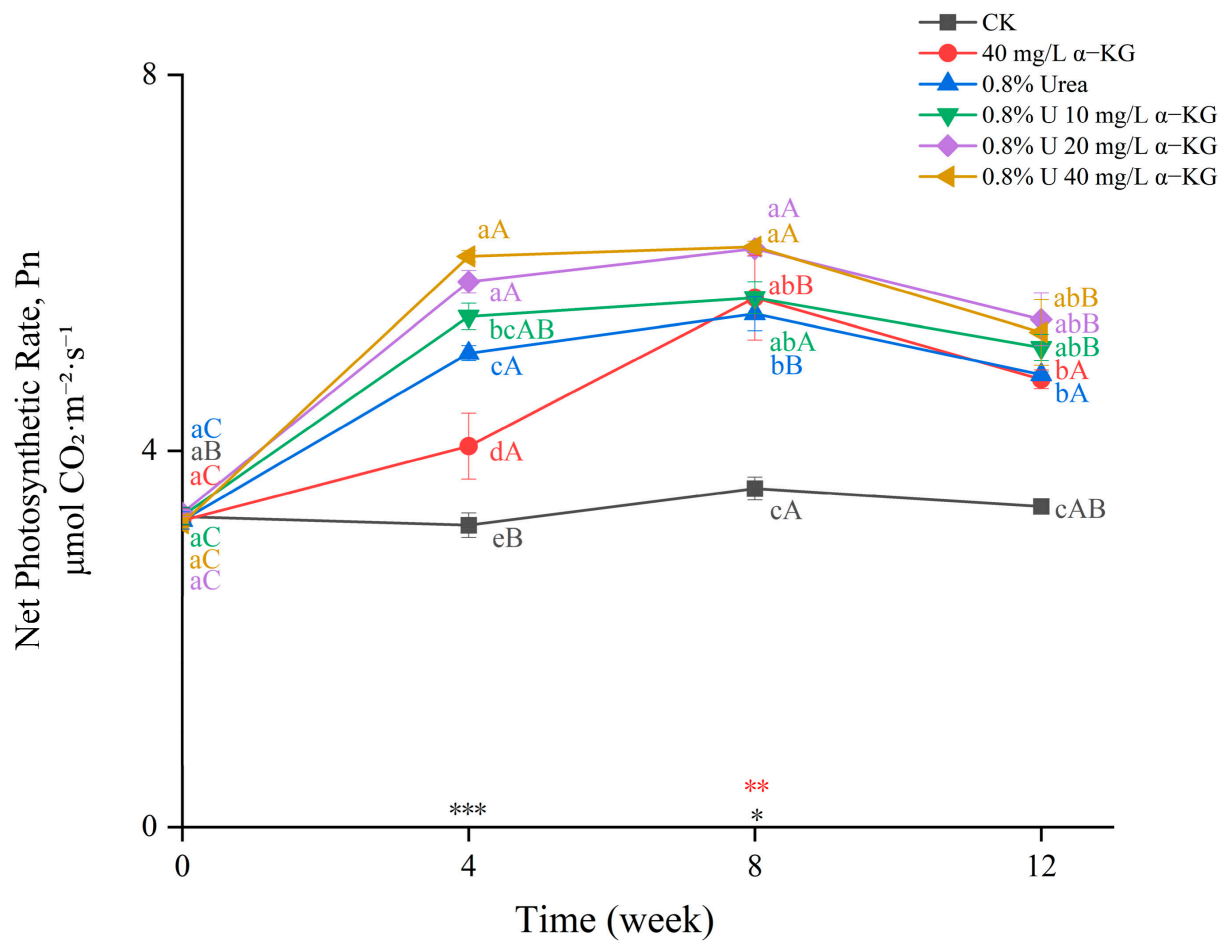
Net photosynthetic rate (*Pn*) of *C. cathayensis* leaves under different treatments at different sampling times. Note: Different lowercase letters indicate significant differences among treatments within the same time point, and different uppercase letters indicate significant differences among time points within the same treatment (two-way ANOVA followed by Tukey’s HSD test, *p* < 0.05). Black asterisks indicate significant linear trends; red asterisks indicate significant quadratic trends across U + α-KG doses (10, 20, 40 mg/L) at each sampling time. * *p* < 0.05, ** *p* < 0.01, *** *p* < 0.001. Error bars represent mean ± SD (*n* = 3). The same note applies to the following figures and tables.

**Figure 5:**
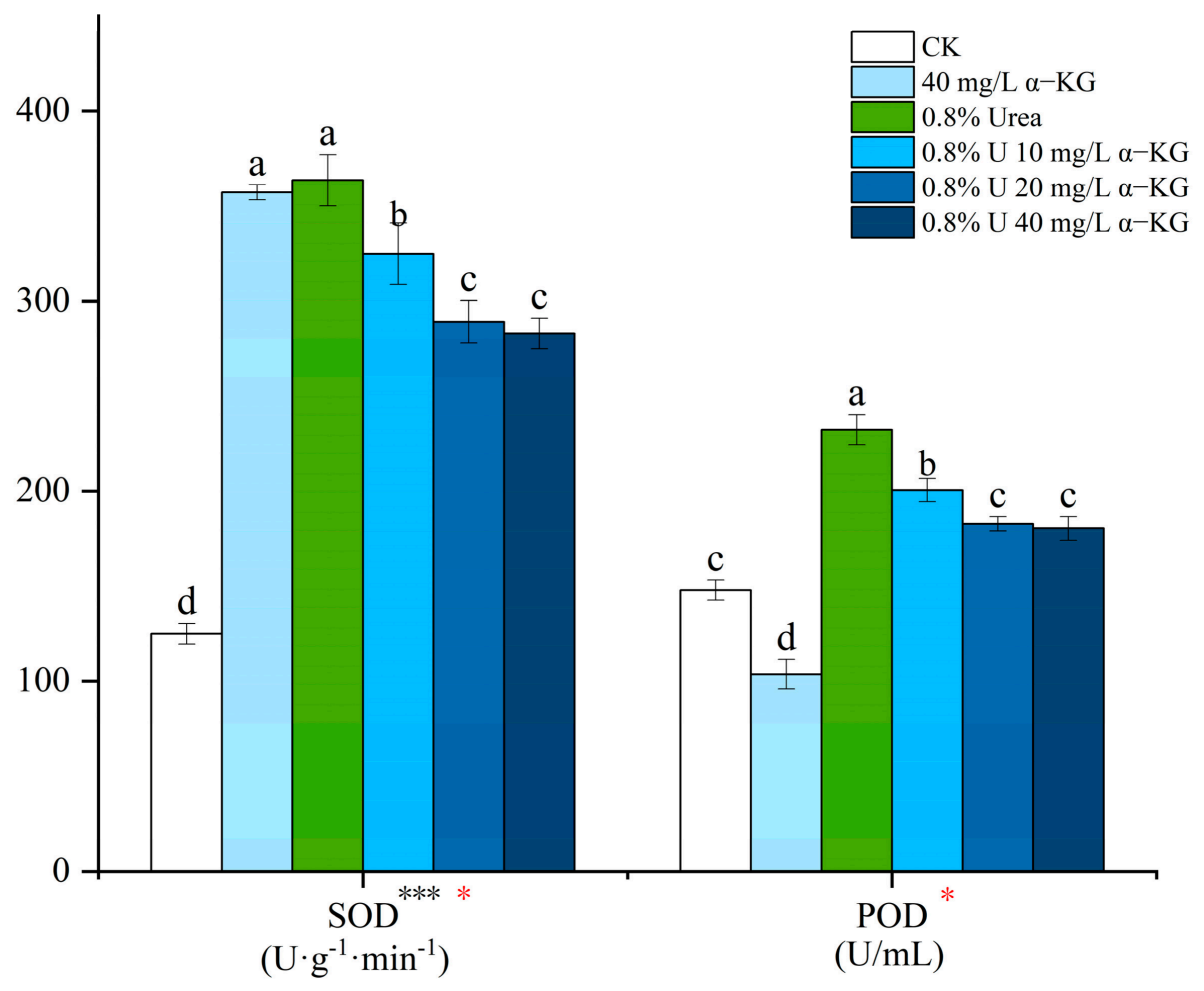
SOD and POD activities in *C. cathayensis* leaves in each group after 12 weeks of treatment. Note: Different letters indicate significant differences among treatments (one-way ANOVA followed by Tukey’s HSD test, *p* < 0.05). Values are means ± SD (*n* = 3). Black asterisks indicate significant linear trends; red asterisks indicate significant quadratic trends across U + α-KG doses (10, 20, 40 mg/L) at each sampling time. * *p* < 0.05, *** *p* < 0.001. The same note applies to the following figures and tables.

**Figure 6:**
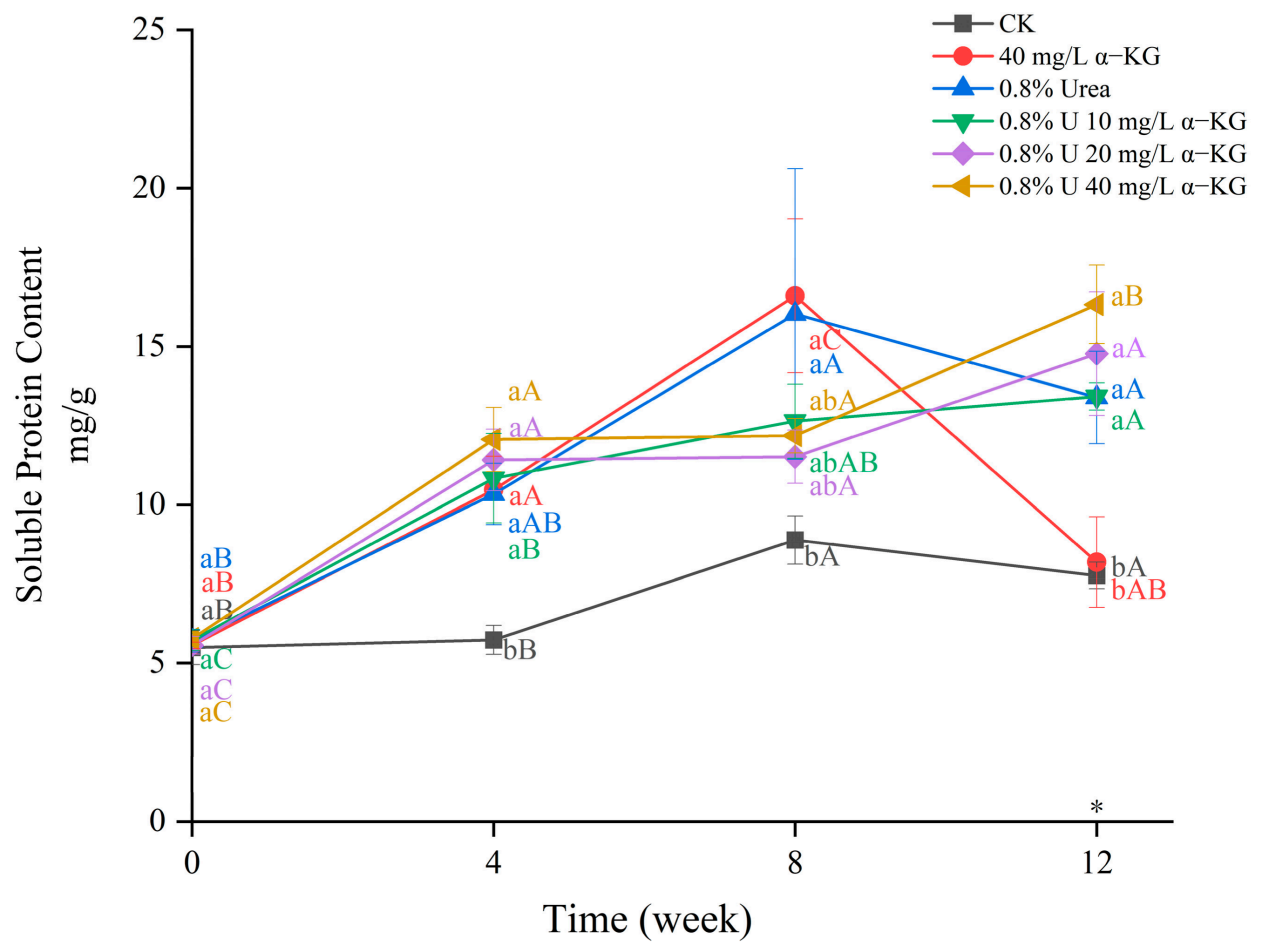
Soluble protein content in leaves of *C. cathayensis* under different treatments at different sampling times. Note: Different lowercase letters indicate significant differences among treatments within the same time point, and different uppercase letters indicate significant differences among time points within the same treatment (two-way ANOVA followed by Tukey’s HSD test, *p* < 0.05). Error bars represent mean ± SD (*n* = 3).

**Figure 7:**
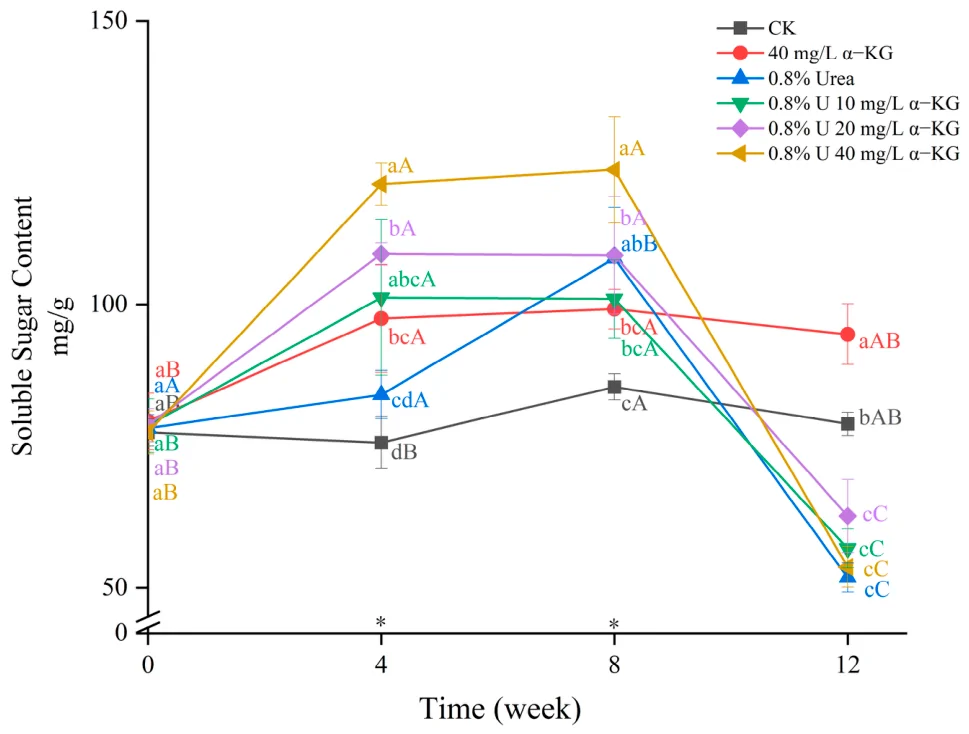
Soluble sugar content in leaves of *C. cathayensis* under different treatments at different sampling times. Note: Different lowercase letters indicate significant differences among treatments within the same time point, and different uppercase letters indicate significant differences among time points within the same treatment (two-way ANOVA followed by Tukey’s HSD test, *p* < 0.05). Black asterisks indicate significant linear trends; * *p* < 0.05. Error bars represent mean ± SD (*n* = 3).

**Figure 8:**
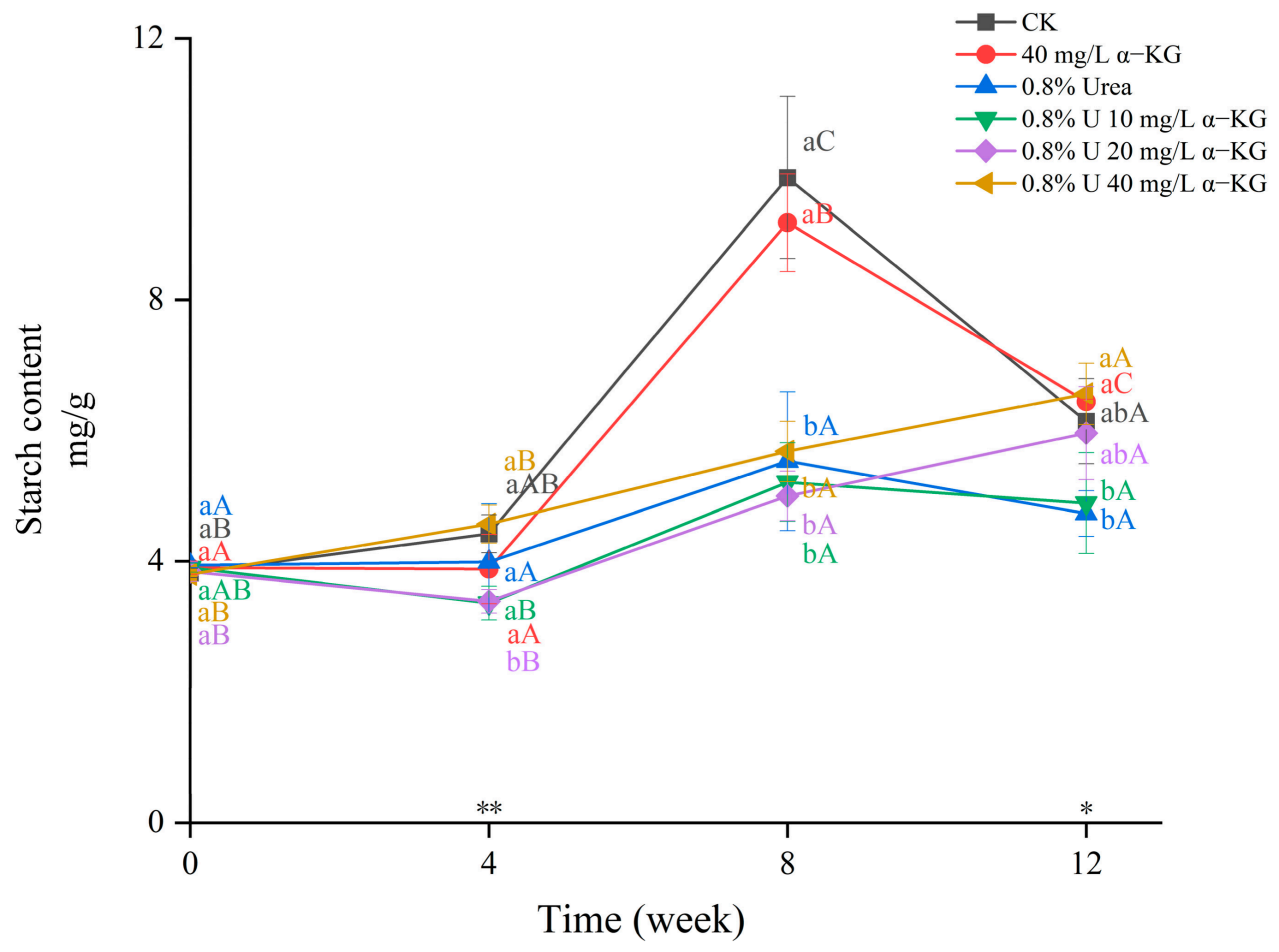
Starch content in leaves of *C. cathayensis* under different treatments at different sampling times. Note: Different lowercase letters indicate significant differences among treatments within the same time point, and different uppercase letters indicate significant differences among time points within the same treatment (two-way ANOVA followed by Tukey’s HSD test, *p* < 0.05). Black asterisks indicate significant linear trends; ** *p* < 0.01. Error bars represent mean ± SD (*n* = 3).

**Figure 9:**
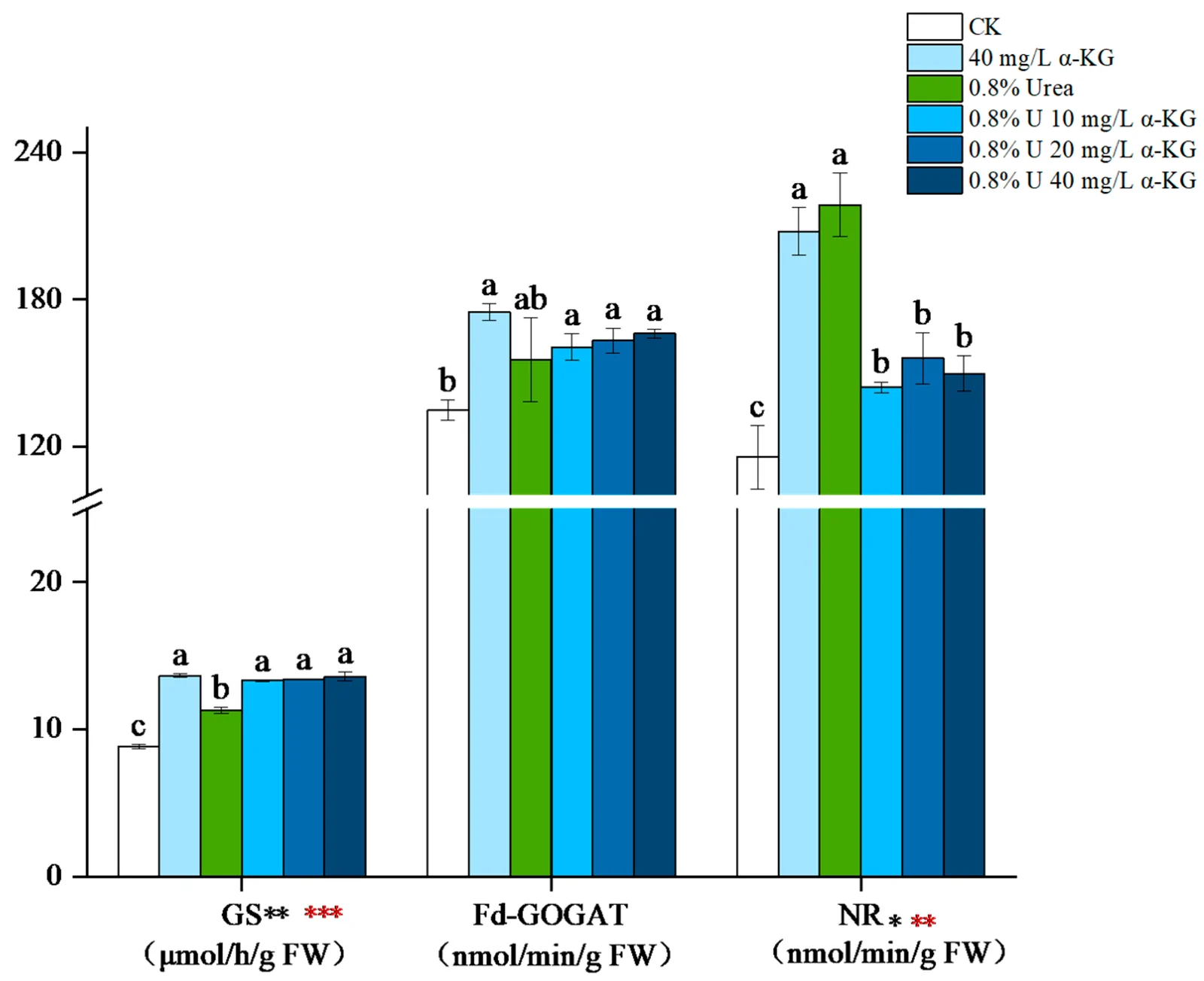
Effects on GS, Fd-GOGAT, and NR activities in *C. cathayensis* leaves after 8 weeks of treatment. Note: Different letters indicate significant differences among treatments (one-way ANOVA followed by Tukey’s HSD test, *p* < 0.05). Values are means ± SD (*n* = 3). Black asterisks indicate significant linear trends; red asterisks indicate significant quadratic trends across U + α-KG doses (10, 20, 40 mg/L) at each sampling time. * *p* < 0.05, ** *p* < 0.01, *** *p* < 0.001. Error bars represent mean ± SD (*n* = 3).

**Figure 10:**
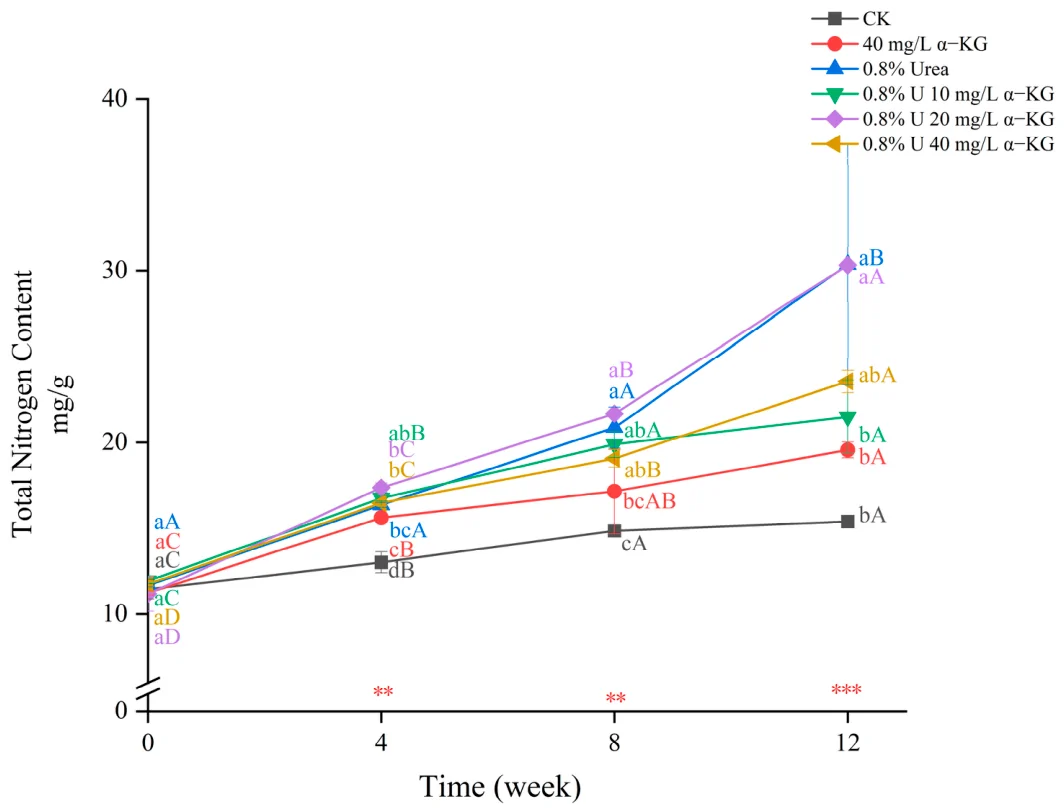
Total nitrogen content in leaves of *C. cathayensis* under different treatments at different sampling times. Note: Different lowercase letters indicate significant differences among treatments within the same time point, and different uppercase letters indicate significant differences among time points within the same treatment (two-way ANOVA followed by Tukey’s HSD test, *p* < 0.05). Red asterisks indicate significant quadratic trends across U + α-KG doses (10, 20, 40 mg/L) at each sampling time. ** *p* < 0.01, *** *p* < 0.001. Error bars represent mean ± SD (*n* = 3).

**Figure 11:**
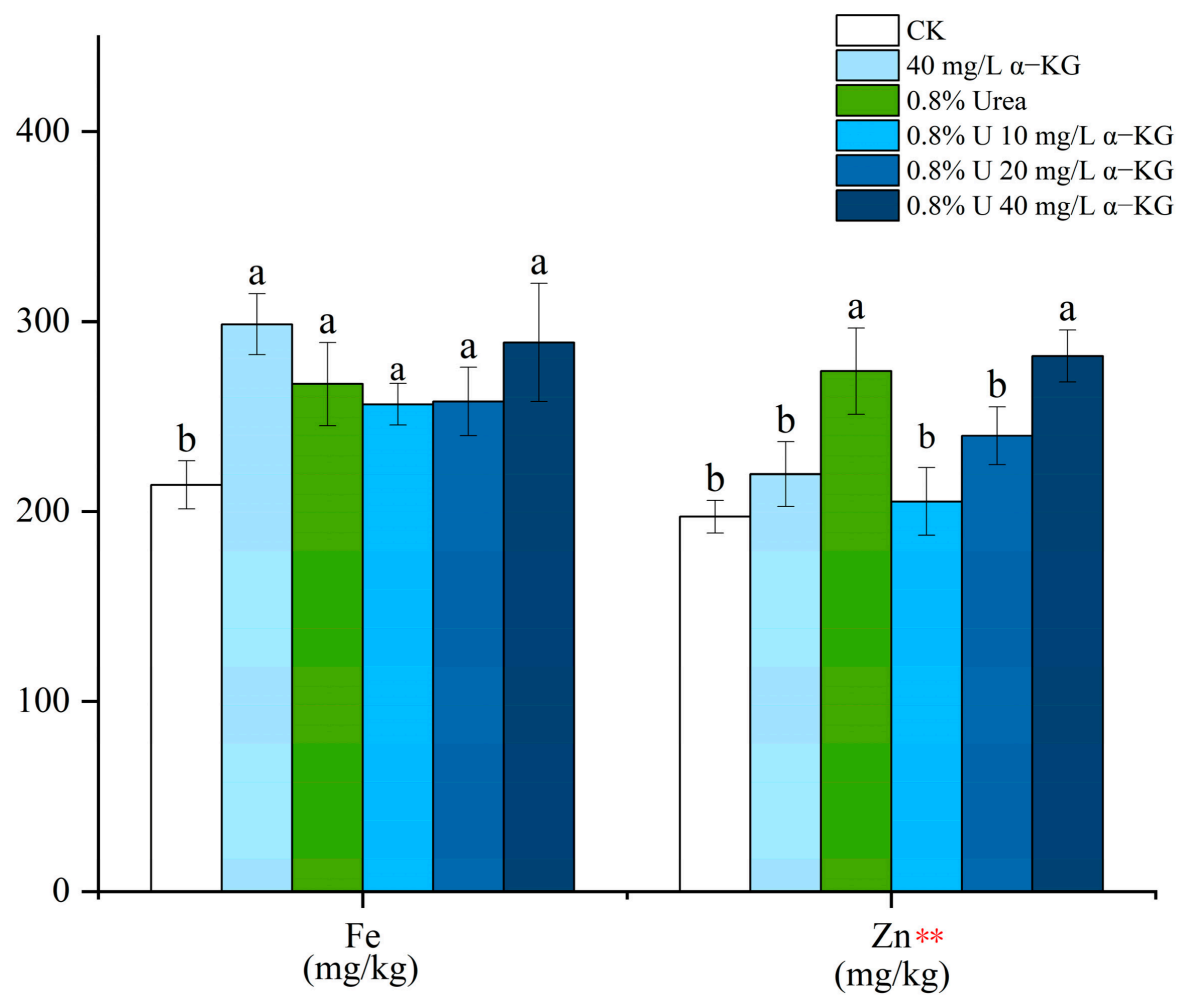
Leaf Fe and Zn concentrations in *C. cathayensis* leaves after 12 weeks of treatment. Different letters indicate significant differences among treatments (one-way ANOVA followed by Tukey’s HSD test, *p* < 0.05). Values are means ± SD (*n* = 3). Red asterisks indicate significant quadratic trends across U + α-KG doses (10, 20, 40 mg/L) at each sampling time. ** *p* < 0.01.

**Table 1:** Experimental treatment design.

Treatment	Urea (*w*/*v*)	α-KG	Description
CK	—	—	Water control
KG3	—	40 mg/L	α-KG alone reference
U	0.8%	—	High urea treatment
UKG1	0.8%	10 mg/L	High urea + low α-KG
UKG2	0.8%	20 mg/L	High urea + medium α-KG
UKG3	0.8%	40 mg/L	High urea + high α-KG

**Table 2:** Photosynthetic pigment contents in leaves of *C. cathayensis* under different treatments at different sampling times.

Group	0 W	4 W	8 W	12 W
	Chlorophyll *a* (μg/mm^2^)			
CK	1.29 ± 0.01 aB	1.55 ± 0.04 bA	1.55 ± 0.08 bA	1.58 ± 0.15 bA
KG3	1.29 ± 0.01 aC	1.68 ± 0.07 aB	2.03 ± 0.12 abA	2.06 ± 0.11 aA
U	1.3 ± 0.01 aC	1.63 ± 0.04 abBC	2.18 ± 0.26 aA	2.02 ± 0.25 aAB
UKG1	1.29 ± 0.01 aC	1.57 ± 0.05 abB	2.16 ± 0.15 aA	2.07 ± 0.05 aA
UKG2	1.3 ± 0.01 aB	1.58 ± 0.06 abB	2.3 ± 0.28 aA	2.14 ± 0.08 aA
UKG3	1.3 ± 0.02 aD	1.63 ± 0.02 abC	2.37 ± 0.07 aA	2.03 ± 0.04 aB
	Chlorophyll *b* (μg/mm^2^)			
CK	0.49 ± 0.01 aA	0.64 ± 0.01 aA	0.51 ± 0.23 aA	0.61 ± 0.06 bA
KG3	0.5 ± 0.01 aC	0.64 ± 0.05 aB	0.53 ± 0.04 aBC	0.86 ± 0.05 abA
U	0.5 ± 0.01 aC	0.68 ± 0.05 aB	0.56 ± 0.07 aBC	0.83 ± 0.07 abA
UKG1	0.51 ± 0.02 aA	0.59 ± 0.11 aA	0.62 ± 0.18 aA	0.82 ± 0.13 abA
UKG2	0.51 ± 0.01 aB	0.61 ± 0.07 aB	0.61 ± 0.01 aB	0.93 ± 0.1 aA
UKG3	0.5 ± 0.01 aB	0.63 ± 0.03 aB	0.58 ± 0.04 aB	1.06 ± 0.13 aA
	Carotenoid (μg/mm^2^)			
CK	0.52 ± 0.01 aB	0.7 ± 0.03 aA	0.41 ± 0.02 bC	0.46 ± 0.04 aBC
KG3	0.53 ± 0.01 aB	0.7 ± 0.07 aA	0.45 ± 0.02 abB	0.47 ± 0.03 aB
U	0.52 ± 0.01 aB	0.69 ± 0.03 aA	0.49 ± 0.05 abB	0.55 ± 0.06 aB
UKG1	0.53 ± 0.01 aA	0.63 ± 0.06 aA	0.58 ± 0.08 aA	0.52 ± 0.02 aA
UKG2	0.52 ± 0.01 aB	0.68 ± 0.06 aA	0.52 ± 0.08 abB	0.53 ± 0.05 aAB
UKG3	0.53 ± 0.01 aA	0.64 ± 0.05 aA	0.51 ± 0.03 abA	0.56 ± 0.09 aA

Note: Different lowercase letters indicate significant differences among treatments within the same sampling time; different uppercase letters indicate significant differences among sampling times within the same treatment (two-way ANOVA followed by Tukey’s HSD test, *p* < 0.05). Values are means ± SD (*n* = 3).

**Table 3:** Chlorophyll fluorescence kinetic parameters of *C. cathayensis* leaves in each group after 8 weeks of treatment.

Group	*ABS/RC*	*φP*_0_	*Ψ*_0_	*φE*_0_	*φD*_0_	*PI_ABS_*
CK	5.268 ± 0.443 a	0.566 ± 0.034 b	0.349 ± 0.051 b	0.198 ± 0.036 b	0.434 ± 0.034 a	0.137 ± 0.046 cd
KG3	4.129 ± 0.530 b	0.664 ± 0.019 a	0.438 ± 0.046 a	0.291 ± 0.039 a	0.336 ± 0.019 b	0.395 ± 0.165 ab
U	5.539 ± 0.301 a	0.535 ± 0.029 b	0.315 ± 0.019 b	0.169 ± 0.010 b	0.465 ± 0.029 a	0.096 ± 0.015 d
UKG1	4.024 ± 0.210 b	0.647 ± 0.021 a	0.445 ± 0.018 a	0.288 ± 0.016 a	0.353 ± 0.021 b	0.371 ± 0.065 bc
UKG2	3.705 ± 0.244 b	0.670 ± 0.005 a	0.471 ± 0.009 a	0.315 ± 0.007 a	0.330 ± 0.005 b	0.490 ± 0.049 ab
UKG3	3.595 ± 0.201 b	0.690 ± 0.003 a	0.496 ± 0.023 a	0.342 ± 0.017 a	0.310 ± 0.003 b	0.614 ± 0.093 a

Note: Different letters indicate significant differences among treatments (one-way ANOVA followed by Tukey’s HSD test, *p* < 0.05). Values are means ± SD (*n* = 3).

## Data Availability

The data presented in this study are available upon request from the corresponding author.

## Supplementary Materials

The following supporting information can be downloaded at: https://www.mdpi.com/article/10.3390/plants15213286/s1, **Supplementary Methods:** Detailed descriptions of plant material and experimental design (S1), growth trait measurements (S2), photosynthetic pigment determination (S3), chlorophyll fluorescence (OJIP) measurement (S4), gas exchange measurement (S5), antioxidant enzyme activity assays (S6), soluble protein/soluble sugar/starch determination (S7), NR/GS/Fd-GOGAT activity assays (S8), total nitrogen content determination (S9), and leaf Fe and Zn content determination (S10). **Table S1.** Experimental treatment schedule and sampling timeline. **Table S2.** Linear and quadratic regression analyses of measured parameters across the α-KG concentration gradient (UKG1–UKG3). **Table S3.** Two-way ANOVA results (treatment × sampling time) for all measured parameters.
