# Supplementary material for: Concentration–Dependent Effects of Exogenous α-Ketoglutarate on PSII Photochemical Performance and C–N Metabolism in Carya cathayensis under High Urea Application
Source: Plants (Basel). 2026 Oct 24;15(21):3286. doi: 10.3390/plants15213286 (PMC13766075; doi:10.3390/plants15213286)
Supplement: Supplementary file 1 [file plants-15-03286-s001.zip › plants-4595054-supplementary.pdf]

Supplementary Methods

S1. Plant Material and Experimental Design

One-year-old healthy Chinese hickory (*Carya cathayensis* Sarg.) seedlings (height approximately 40–50 cm, basal diameter 6–8 mm) were selected as plant material. Seedlings were grown in non-woven fabric bags (20 cm diameter × 15 cm height). The substrate was provided by Hangzhou Jinhai Agricultural Technology Co., Ltd.; the detailed composition is proprietary. The initial physicochemical properties of the substrate were not measured, but all plants used the same substrate. Seedlings were acclimated in a greenhouse for 4 weeks before the experiment.

Six treatments were established: water control (CK), α-ketoglutarate alone at 40 mg/L (KG3), 0.8% urea alone (U), and urea combined with 10, 20, or 40 mg/L α-ketoglutarate (UKG1, UKG2, UKG3, respectively). Fifteen seedlings per treatment were arranged randomly. Foliar spraying was applied until leaves were uniformly wetted without dripping (approximately 35 mL per plant). On day 1 of each cycle, 0.8% (w/v) urea was sprayed; 72 h later (day 4), α-ketoglutarate (or deionized water for controls) was sprayed. Each cycle lasted 7 days, and treatments continued for 12 weeks. Week 0 (baseline) samples were collected from additional plants. At Weeks 4, 8, and 12, the 3rd–4th fully expanded compound leaves from the shoot apex were sampled 4 days after α-ketoglutarate spraying. Samples were immediately frozen in liquid nitrogen and stored at −80 °C. Three biological replicates were used per treatment per time point.

S2. Growth Trait Measurements

Five healthy leaves per treatment were selected to measure specific leaf area (SLA) using a YMJ-CHA3 intelligent leaf area meter. Leaf area (S_1, cm²) and dry mass (W_2, mg) were recorded: SLA = $S_1/W_2 \times 1000$ (cm²/g). Five healthy leaves per treatment were selected to measure leaf dry matter content (LDMC). Fresh mass (W_1, g) and dry mass (W_2, mg) were recorded: LDMC = W_2/W_1 (mg/g).

S3. Photosynthetic Pigment Determination

Leaf discs (6 mm diameter) were punched and immediately immersed in 10 mL of 95% ethanol in the dark for 48 h. The supernatant was mixed, and absorbance was measured at 470, 649, and 665 nm. Chlorophyll a (Chl a), chlorophyll b (Chl b), and carotenoid (C_{x+c}) contents were calculated according to the modified Arnon equations of Lichtenthaler and Wellburn:

$$C_a = 13.95\ A_{665} - 6.88\ A_{649};$$

$$Cb = 24.96\ A_{649} - 7.32\ A_{665};$$

$$C_{x+c} = (1000\ A_{470} - 2.05\ C_a - 114.8\ Cb) / 245$$

Pigment contents are expressed per unit leaf area (μg/mm²).

S4. Chlorophyll Fluorescence (OJIP) Measurement

At Week 8, chlorophyll a fluorescence induction kinetics were measured using a YZQ-500 modulated chlorophyll fluorometer (Yizongqi Technology Co., Ltd., Beijing, China). Leaves

were dark-adapted for 30 min with leaf clips, then exposed to saturating blue light at 3000 μmol photons·m^{-2}·s^{-1} for 5 s (saturating pulse duration, 800 ms). Fluorescence values were recorded at 50 μs (F_o), 300 μs, 2 ms (FJ), 30 ms (FI), and maximum fluorescence (Fm). JIP-test parameters were derived according to Strasser et al. [34,35] as follows:

$VJ = (FJ - F_o)/(Fm - F_o)$ —— relative variable fluorescence at the J step

$M_o = 4(F_{300}μs - F_o)/(Fm - F_o)$ —— initial slope of the fluorescence rise

$φP_o = TR_o/ABS = (Fm - F_o)/Fm$ —— maximum quantum yield of primary photochemistry (i.e., Fv/Fm)

$ψ_o = ET_o/TR_o = 1 - VJ$ —— probability that a trapped exciton moves an electron further than QA^-

$φE_o = ET_o/ABS = φP_o \times ψ_o$ —— quantum yield of electron transport

$φD_o = F_o/Fm = 1 - φP_o$ —— quantum yield of energy dissipation

$ABS/RC = M_o \times (1/VJ) \times (1/φP_o)$ —— apparent absorbed flux per QA-reducing PSII reaction center

$PIABS = (RC/ABS) \times [φP_o/(1-φP_o)] \times [ψ_o/(1-ψ_o)]$ —— performance index on an absorption basis

S5. Gas Exchange Measurement

Net photosynthetic rate (Pn) was measured using an LI-6800 portable photosynthesis system (LI-COR, Lincoln, NE, USA). Measurement conditions: CO_2 concentration 400 μmol/mol, leaf temperature 25 °C, red/blue light ratio 9:1, photosynthetically active radiation 1200 μmol·m^{-2}·s^{-1}, airflow 500 μmol/s, relative humidity 50%. Measurements were taken from 8:30 to 16:30.

S6. Antioxidant Enzyme Activity Assays

Fresh leaf tissue (0.5 g) was homogenized in 0.5 mL of pre-cooled extraction medium (50 mmol/L phosphate buffer, pH 7.8) using a high-throughput tissue grinder. The homogenate was brought to 5 mL with extraction medium and centrifuged at 6000 rpm for 30 min at 4 °C. The supernatant was used for SOD, POD, and soluble protein assays.

SOD activity was determined by the nitro blue tetrazolium (NBT) reduction method. The reaction mixture (3.3 mL) contained 1.5 mL of 50 mmol/L phosphate buffer (pH 7.8), 0.3 mL of 130 mmol/L methionine, 0.3 mL of 750 μmol/L NBT, 0.3 mL of 100 μmol/L EDTA-Na_2, 0.5 mL of distilled water, 0.3 mL of 20 μmol/L riboflavin, and 0.1 mL of enzyme extract. The control tube used buffer instead of enzyme extract. Tubes were illuminated at 25 °C under 4000 lx for 10 min, then immediately placed in the dark to terminate the reaction. Absorbance was measured at 560 nm against a darkened control. SOD activity was calculated as:

$SOD\ activity = (A_o - As) \times Vt / (0.5 \times A_o \times Wf \times Vi)$

where A_0 is the absorbance of the illuminated control, As is the absorbance of the sample, Vt is the total extract volume (mL), Vi is the sample volume used in the assay (mL), and Wf is the fresh sample mass (g).

POD activity was determined by the guaiacol oxidation method. A 3 mL aliquot of POD reaction solution was placed in a quartz cuvette, 0.1 mL of enzyme extract was added, and the change in absorbance at 470 nm was recorded over 3 min. POD activity was calculated as:

$$\text{POD activity} = (\Delta A_{470} \times Vt) / (0.001 \times Wf \times Vs \times t)$$

where ΔA_{470} is the absorbance change over the reaction time, Vt is the total enzyme extract volume (mL), Vs is the enzyme volume used in the assay (mL), Wf is the fresh sample mass (g), and t is the reaction time (min).

S7. Soluble Protein, Soluble Sugar, and Starch Contents

Soluble protein was determined by the Coomassie Brilliant Blue G-250 method. A standard curve was prepared using bovine serum albumin (0–100 μg). A 0.1 mL aliquot of sample extract was mixed with 0.9 mL of distilled water and 5 mL of Coomassie Brilliant Blue G-250 reagent, allowed to stand for 2 min, and absorbance was measured at 595 nm. Soluble protein content was calculated as:

$$\text{Soluble protein (mg/g)} = (C \times Vt) / (Vs \times Wf \times 1000)$$

where C is the protein amount read from the standard curve (μg), Vt is the total extract volume (mL), Vs is the sample volume used (mL), and Wf is the fresh sample mass (g). Standard curve: $y = 0.0053x + 0.0116$, $R^2 = 0.9965$.

Dried leaf powder (100 mg) was mixed with 2 mL of distilled water, boiled in a water bath for 30 min, cooled on ice, and centrifuged at 5000 rpm for 15 min. The supernatant was collected; the extraction was repeated once, and the supernatants were combined and brought to 10 mL. A 1 mL aliquot was mixed with 5 mL of anthrone reagent, boiled for 10 min, cooled on ice, and absorbance was measured at 620 nm. A standard curve was prepared using glucose standard solution (0–100 μg).

Starch content was determined as follows: the residue remaining after soluble sugar extraction was mixed with 1 mL of pre-cooled 52% perchloric acid, extracted at 25 °C with shaking at 100 rpm for 30 min, and centrifuged at 5000 rpm for 15 min. The supernatant was collected; extraction was repeated once, and supernatants were combined and brought to 10 mL. The subsequent anthrone-sulfuric acid colorimetric procedure was the same as above. Starch content is expressed as mg glucose equivalent per g dry mass: $W = C \times Vt / (Vs \times Wd \times 10^3) \times 0.9$ (where 0.9 is the conversion factor). Standard curve: $y = 0.0094x + 0.0079$, $R^2 = 0.9978$.

S8. NR, GS, and Fd-GOGAT Activities

Nitrate reductase (NR), glutamine synthetase (GS), and ferredoxin-dependent glutamate synthase (Fd-GOGAT) activities were measured using commercial assay kits (Suzhou Mengxi Biotechnology Co., Ltd., Suzhou, China; NR cat. no. M0203A, GS cat. no. M0201A, Fd-GOGAT cat. no. M0206A) according to the manufacturer's instructions.

S9. Total Nitrogen Content

Total nitrogen was determined by the Kjeldahl method. Dried leaf powder (0.3–0.5 g, passed through a 0.25–0.5 mm sieve) was digested with 5 mL of concentrated sulfuric acid; 2 mL of hydrogen peroxide was added in two portions during digestion, and digestion continued until the solution became clear and colorless. After cooling, the digest was transferred and brought to 100 mL. For distillation, 30 mL of boric acid was used as the receiving solution, 20 mL of 400 g/L sodium hydroxide was added, and titration was performed with standard acid.

S10. Leaf Fe and Zn Contents

Leaf Fe and Zn contents were measured by inductively coupled plasma optical emission spectrometry (ICP-OES; Prodigy7, Leeman, Hudson, NH, USA), following GB 5009.268-2016, National Food Safety Standard—Determination of Multi-Elements in Foods [41]. Leaf samples were not washed before drying and digestion. Dried leaf powder (0.5 g, accurate to 0.0001 g) was weighed into a PTFE digestion vessel, soaked overnight in 5 mL of nitric acid, and digested in a microwave digestion system (MARS6, CEM, Matthews, NC, USA) with the following temperature program: 80 °C for 1–2 h, 120 °C for 1–2 h, then ramped to 160 °C and held for 4 h. After natural cooling, the vessel was opened, the acid was evaporated to near dryness, and the digest was transferred to a 25 mL volumetric flask and brought to volume with 1% nitric acid. A reagent blank was prepared simultaneously. Element content was calculated as $C_1 \times V_1 / m$, expressed on a dry-weight basis (mg/kg), where C_1 is the measured element concentration (mg/L), V_1 is the final digest volume (mL), and m is the sample mass (g).

Table S1. Treatment schedule.

Jun 15, 2022	Jul 15, 2022	Jul 17, 2022 9:00 AM	Jul 20, 20229:00 AM		
Acclimation	(W0 sampling)	Jul 24, 2022 9:00 AM	Jul 27, 2022 9:00 AM		
		Jul 31, 2022 9:00 AM	Aug 3, 2022 9:00 AM		
		Aug 7, 2022 9:00 AM	Aug 10, 2022 9:00 AM	Aug 13, 2022 9:00 AM	Week 4 sampling
		Aug 14, 2022 9:00 AM	Aug 17, 2022 9:00 AM		
		Aug 21, 2022 9:00 AM	Aug 24, 2022 9:00 AM		
		Aug 28, 2022 9:00 AM	Aug 31, 2022 9:00 AM		
		Sep 4, 2022 9:00 AM	Sep 7, 2022 9:00 AM	Sep 10, 2022 9:00 AM	Week 8 sampling
		Sep 11, 2022 9:00 AM	Sep 14, 2022 9:00 AM		
		Sep 18, 2022 9:00 AM	Sep 21, 2022 9:00 AM		
		Sep 25, 2022 9:00 AM	Sep 28, 2022 9:00 AM		
		Oct 2, 2022 9:00 AM	Oct 5, 2022 9:00 AM	Oct 8, 2022 9:00 AM	Week 12 sampling
	35 mL per plant				
	CK	Spray UP H_2O	Spray UP H_2O		
	KG3	Spray UP H_2O	40 mg/L α-KG		
	U	0.8% Urea	Spray H_2O		
	UKG1	0.8% Urea	10 mg/L α-KG		
	UKG2	0.8% Urea	20 mg/L α-KG		
	UKG3	0.8% Urea	40 mg/L α-KG		

Table S2. Linear and quadratic regression analyses of measured parameters across the α-KG concentration gradient (UKG1–UKG3).

Parameter	Time	Linear slope	Linear P	Quadratic P
LDMC	W0	0.057	0.362	0.52
LDMC	W4	0.398	0.495	0.119
LDMC	W8	-0.181	0.749	0.274
LDMC	W12	1.133	0.123	0.502
SLA	W0	0	0.726	0.859
SLA	W4	0.001	0.15	0.834
SLA	W8	0	0.775	0.233
SLA	W12	-0.001	0.003 **	0.535

Soluble protein	W0	0.003	0.68	0.424
Soluble protein	W4	0.039	0.209	0.844
Soluble protein	W8	-0.009	0.748	0.18
Soluble protein	W12	0.094	0.028 *	0.709
Starch	W0	-0.003	0.232	0.721
Starch	W4	0.043	0.001 **	0.082
Starch	W8	0.018	0.208	0.344
Starch	W12	0.052	0.023 *	0.326
Total N	W0	-0.002	0.932	0.156
Total N	W4	-0.014	0.237	0.004 **
Total N	W8	-0.042	0.231	0.002 **
Total N	W12	0.011	0.931	<0.001 ***
Soluble sugar	W0	-0.044	0.661	0.87
Soluble sugar	W4	0.658	0.015 *	0.868
Soluble sugar	W8	0.756	0.011 *	0.985
Soluble sugar	W12	-0.158	0.328	0.09
Chl a	W0	0	0.566	0.539
Chl a	W4	0.002	0.128	0.664
Chl a	W8	0.007	0.21	0.613
Chl a	W12	-0.002	0.356	0.091
Chl b	W0	0	0.675	0.909
Chl b	W4	0.001	0.501	0.982
Chl b	W8	-0.001	0.665	0.958
Chl b	W12	0.008	0.039 *	0.798
Carotenoids	W0	0	0.992	0.128
Carotenoids	W4	0	0.865	0.328

Carotenoids	W8	-0.002	0.284	0.513
Carotenoids	W12	0.001	0.436	0.967
Pn (net photosynthesis)	W0	-0.003	0.127	0.217
Pn (net photosynthesis)	W4	0.02	<0.001 ***	0.106
Pn (net photosynthesis)	W8	0.016	0.025 *	0.006 **
Pn (net photosynthesis)	W12	0.004	0.624	0.26
Height increment	—	0.413	0.097	0.008 **
NR activity	—	-1.311	0.029 *	0.007 **
GOGAT activity	—	0.253	0.158	0.653
GS activity	—	0.047	0.005 **	<0.001 ***
ABS/RC	—	-0.042	0.003 **	<0.001 ***
φP_0 (TRo/ABS)	—	0.003	0.001 **	<0.001 ***
Ψ_0 (ETo/TRo)	—	0.004	0.001 **	<0.001 ***
$\varphi E0$ (ETo/ABS)	—	0.004	<0.001 ***	<0.001 ***
φD_0 (Fo/Fm)	—	-0.003	0.001 **	<0.001 ***
PI_{ABS}	—	0.012	<0.001 ***	0.005 **
Fe content	—	0.629	0.170	0.159
Zn content	—	0.695	0.334	0.010 **
SOD activity	—	-1.953	0.001 ***	0.011 *
POD activity	—	0.62	0.172	0.047 *

Note: * $p < 0.05$, ** $p < 0.01$, *** $p < 0.001$. Linear slope represents the change in response per 1 mg/L increase in α-KG concentration. Time points marked with "—" indicate measurements taken only at week 8 or week12. (one-time sampling).

Table S3. Two-way ANOVA results (treatment × sampling time) for all measured parameters.

Parameter	Effect	df	F	P	partial η^2
LDMC (mg/g)	Treatment	5, 48	16.32	<0.001	0.630

LDMC (mg/g)	Time	3, 48	420.55	<0.001	0.963
LDMC (mg/g)	T × Time	15, 48	5.22	<0.001	0.620
SLA (cm^2/g)	Treatment	5, 48	33.99	<0.001	0.780
SLA (cm^2/g)	Time	3, 48	418.73	<0.001	0.963
SLA (cm^2/g)	T × Time	15, 48	7.25	<0.001	0.694
Soluble protein (mg/g)	Treatment	5, 48	17.60	<0.001	0.647
Soluble protein (mg/g)	Time	3, 48	101.77	<0.001	0.864
Soluble protein (mg/g)	T × Time	15, 48	7.47	<0.001	0.700
Starch (mg/g)	Treatment	5, 48	21.46	<0.001	0.691
Starch (mg/g)	Time	3, 48	121.35	<0.001	0.884
Starch (mg/g)	T × Time	15, 48	11.60	<0.001	0.784
Total N (mg/g)	Treatment	5, 48	26.31	<0.001	0.733
Total N (mg/g)	Time	3, 48	168.12	<0.001	0.913
Total N (mg/g)	T × Time	15, 48	7.78	<0.001	0.709
Soluble sugar (mg/g)	Treatment	5, 48	13.05	<0.001	0.576
Soluble sugar (mg/g)	Time	3, 48	154.22	<0.001	0.906
Soluble sugar (mg/g)	T × Time	15, 48	15.73	<0.001	0.831
Chl a **(**μg/mm²**)**	Treatment	5, 48	15.12	<0.001	0.612
Chl a **(**μg/mm²**)**	Time	3, 48	185.43	<0.001	0.921
Chl a **(**μg/mm²**)**	T × Time	15, 48	5.02	<0.001	0.611
Chl b **(**μg/mm²**)**	Treatment	5, 48	3.10	0.017	0.244
Chl b **(**μg/mm²**)**	Time	3, 48	57.09	<0.001	0.781
Chl b **(**μg/mm²**)**	T × Time	15, 48	2.31	0.014	0.419
Carotenoids **(**μg/mm²**)**	Treatment	5, 48	1.69	0.155	0.150
Carotenoids **(**μg/mm²**)**	Time	3, 48	56.83	<0.001	0.780
Carotenoids **(**μg/mm²**)**	T × Time	15, 48	2.25	0.017	0.413

Pn (μmol $CO_2 \cdot m^{-2} \cdot s^{-1}$)	Treatment	5, 48	178.50	<0.001	0.949
Pn (μmol $CO_2 \cdot m^{-2} \cdot s^{-1}$)	Time	3, 48	511.79	<0.001	0.970
Pn (μmol $CO_2 \cdot m^{-2} \cdot s^{-1}$)	T × Time	15, 48	27.16	<0.001	0.895

Note: df, degrees of freedom; F, F-statistic; P, p-value; partial η^2, partial eta squared. NS, not significant ($p > 0.05$). T × Time, interaction between treatment and sampling time.
